# Amygdala electrical-finger-print (AmygEFP) NeuroFeedback guided by individually-tailored Trauma script for post-traumatic stress disorder: Proof-of-concept

**DOI:** 10.1016/j.nicl.2021.102859

**Published:** 2021-10-15

**Authors:** Tom Fruchtman-Steinbok, Jackob N. Keynan, Avihay Cohen, Iman Jaljuli, Shiri Mermelstein, Gadi Drori, Efrat Routledge, Michael Krasnoshtein, Rebecca Playle, David E.J. Linden, Talma Hendler

**Affiliations:** a Sagol Brain Institute, Tel-Aviv Medical Center, Tel-Aviv, Israel; b School of Psychological Sciences, Gershon H. Gordon Faculty of Social Sciences, Tel-Aviv University, Tel-Aviv, Israel; c Sagol School of Neuroscience, Tel-Aviv University, Tel-Aviv, Israel; d Sackler Faculty of Medicine, Tel-Aviv University, Tel-Aviv, Israel; e Department of Statistics and Operations Research, School of Mathematical Sciences, Tel-Aviv University, Tel-Aviv, Israel; f Psychiatric Department, Tel-Aviv Sourasky Medical Center, Tel-Aviv, Israel; g Department of Psychiatry & Behavioral Science, Stanford University School of Medicine, Stanford, CA, USA; h Center for Trials Research, College of Biomedical & Life Sciences, Cardiff University, Cardiff, UK; i Division of Psychological Medicine and Clinical Neurosciences, School of Medicine, Cardiff University, Cardiff, UK

**Keywords:** Self-regulation, Limbic activity, Neuromodulation, fMRI, EEG

## Abstract

**Background:**

Amygdala activity dysregulation plays a central role in post-traumatic stress disorder (PTSD). Hence learning to self-regulate one's amygdala activity may facilitate recovery. PTSD is further characterized by abnormal contextual processing related to the traumatic memory. Therefore, provoking the personal traumatic narrative while training amygdala down-regulation could enhance clinical efficacy. We report the results of a randomized controlled trial (NCT02544971) of a novel self-neuromodulation procedure (i.e. NeuroFeedback) for PTSD, aimed at down-regulating limbic activity while receiving feedback from an auditory script of a personal traumatic narrative. To scale-up applicability, neural activity was probed by an fMRI-informed EEG model of amygdala activity, termed Amygdala Electrical Finger-Print (AmygEFP).

**Methods:**

Fifty-nine adults meeting DSM-5 criteria for PTSD were randomized between three groups: Trauma-script feedback interface (Trauma-NF) or Neutral feedback interface (Neutral-NF), and a control group of No-NF (to control for spontaneous recovery). Before and immediately after 15 NF training sessions patients were blindly assessed for PTSD symptoms and underwent one session of amygdala fMRI-NF for transferability testing. Follow-up clinical assessment was performed at 3- and 6-months following NF treatment.

**Results:**

Patients in both NF groups learned to volitionally down-regulate AmygEFP signal and demonstrated a greater reduction in PTSD symptoms and improved down-regulation of the amygdala during fMRI-NF, compared to the No-NF group. The Trauma-NF group presented the largest immediate clinical improvement.

**Conclusions:**

This proof-of-concept study indicates the feasibility of the AmygEFP-NF process-driven as a scalable intervention for PTSD and illustrates its clinical potential. Further investigation is warranted to elucidate the contribution of AmygEFP-NF beyond exposure and placebo effects.

## Highlights

• Randomized clinical trial with a novel self-neuromodulation training in PTSD.
• Demonstration of feasibility of an fMRI-informed EEG model of Amygdala modulation (AmygEFP).
• Individually-tailored trauma-related content as the training feedback interface.
• Results showed reduction of PTSD symptoms following AmygEFP trauma-related feedback training.

## 1 Introduction

Post-Traumatic Stress Disorder (PTSD), is a chronic and debilitating condition (Benjet, 2016, Karam, 2014), affecting approximately 15% of trauma-exposed individuals (Breslau et al., 1991, Santiago et al., 2013). Animal models and human brain research point to amygdala hyperactivity as a core neural abnormality in PTSD (Fenster et al., 2018, Hayes et al., 2012, Lanius, 2010, Shin et al., 2006). Prospective functional magnetic-resonance-imaging (fMRI) studies demonstrated the pivotal role of the amygdala in the dynamic of traumatic stress, by showing that heightened amygdala reactivity prior to, or immediately after, traumatic exposure corresponded to higher PTSD severity following it (Admon, 2009, Admon et al., 2013, Stevens, 2017). Therefore, learning to regulate one's own amygdala activity may minimize detrimental processes and facilitate recovery ones. Such regulation of amygdala activity could be obtained volitionally via a closed-loop brain-computer-interface guided procedure of reinforcement learning termed fMRI-neurofeedback (fMRI-NF) (Young, 2017, Paret et al., 2014, Paret et al., 2016, Zotev et al., 2011, Herwig et al., 2019, Sitaram, 2017).

**Fig. 1:**
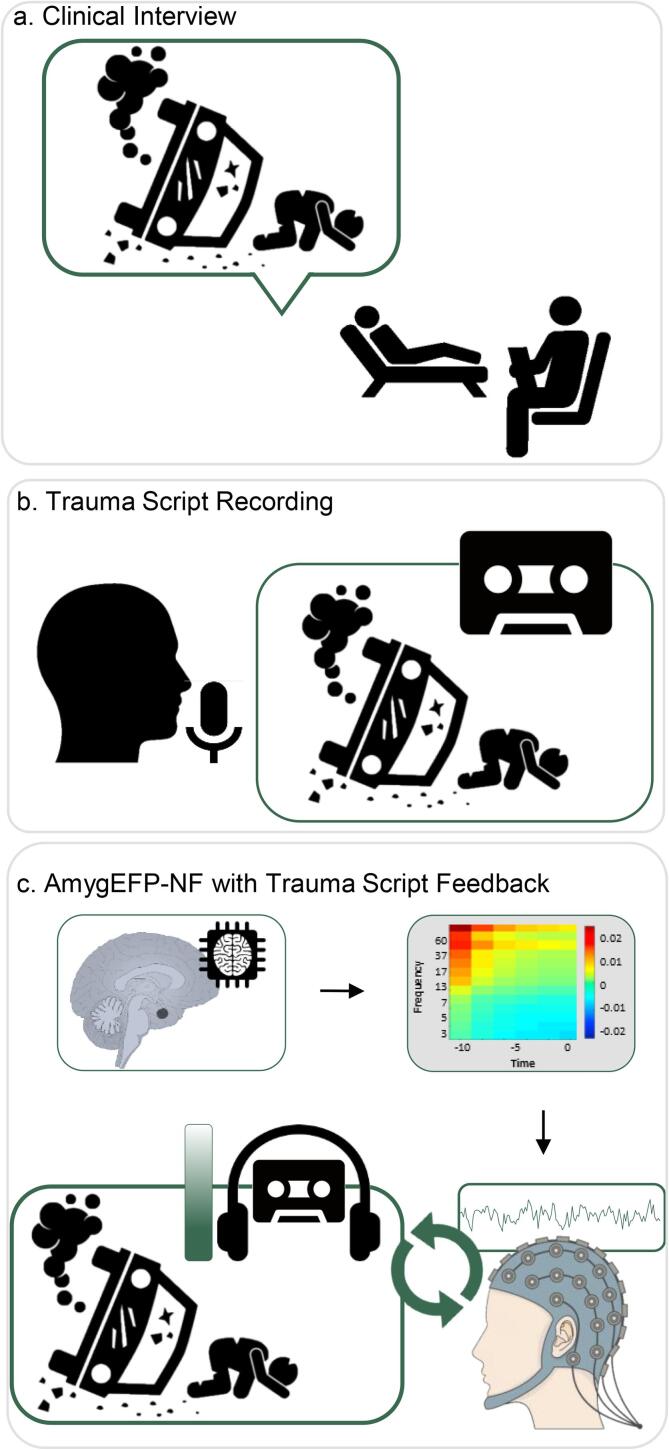
**AmygEFP-NF with Individual Trauma Script Feedback. a. Clinical Interview.** During the first trauma NF session patients were interviewed about the traumatic event that met DSM-5 criterion A in order to produce a scripted detailed chain of events, including thoughts, feelings, sensations and contextual information. **b. Trauma Script Recording.** The interview was edited and then recorded as a three-minute audio segment (second-person male voice in present tense). **c. AmygEFP-NF with Individual Trauma Script Feedback.** Each patient trained using Amygdala Electrical-Fingerprint (AmygEFP-NF) with a personalized trauma-narrative feedback. The feedback indicating AmygEFP signal was the volume of the trauma-narrative recording. A successful reduction of AmygEFP signal reduced the volume of the trauma-narrative.

PTSD is characterized by abnormal contextual processing related to the traumatic event narrative (Shalev et al., 2017). Accordingly, several behavioral therapies for PTSD focus on processing the traumatic memory, the most prominent of which is prolonged exposure therapy (PE) (Foa, 2011). A recent 'process-based' perspective for NF suggested that clinical efficacy in psychiatry could be increased by coupling volitional neural regulation with activation of disorder-specific cognitive-affective processes (Lubianiker et al., 2019). Following this suggestion and building upon the vast evidence on abnormal memory processing in PTSD (Foa, 2011), we propose that amygdala-NF for PTSD would be more effective when reinstating the traumatic memory while practicing amygdala down-regulation. Accordingly, the current study introduces a novel NF training approach for PTSD in which the feedback interface consists of an individually-tailored audio script of the personal traumatic narrative (Fig. 1). Preliminary evidence suggests that PTSD patients can learn amygdala self-regulation through fMRI-NF while confronted with trauma reminders. A feasibility study (n = 3) (Gerin et al., 2016) showed successful down-regulation of amygdala BOLD activity in patients, *after* listening to a recording of their traumatic memory; two out of three exhibited reduced PTSD severity. Nicholson et al. (Nicholson, 2017), similarly showed that PTSD patients (n = 10) learned amygdala down-regulation while viewing trauma-related words. While these studies made important initial steps to include disorder-specific content in NF treatment of PTSD, the traumatic content was only incorporated as single words rather than the full narrative, or not fully coupled with amygdala regulation. That is, appearing between epochs of regulation-training (Keynan, 2016) and not integrated into the feedback itself. Furthermore, by relying on fMRI-NF, the scalability of these NF interventions is substantially limited.

To improve treatment scalability (accessibility, mobility, and cost-effectiveness), while maintaining precise targeting of a specific neural mechanism, NF in the current study was guided by a previously developed fMRI-informed EEG model of amygdala activity (See Supplementary Fig. S1). This method, termed Electrical-Finger-Print (EFP) (Meir-Hasson et al., 2014, Meir-Hasson et al., 2016), was validated as an amygdala-BOLD proxy (AmygEFP) (Keynan, 2016) and its efficacy as a self-neuromodulation tool for improved emotion regulation was recently demonstrated in healthy soldiers under ongoing chronic military stress (Keynan, 2019).

The current proof-of-concept study presents a novel NF approach for PTSD by addressing two major methodological issues: improving process-precision by integrating individually tailored disorder-specific content as the reinforcing feedback interface, and enhancing scalability by using AmygEFP to probe amygdala down-regulation (Fig. 1). Our goals were to (a) Test whether chronic PTSD patients are capable of regulating AmygEFP by undergoing 15 NF training sessions. (b) Test whether AmygEFP-NF guided by the individually tailored traumatic narrative (Trauma-NF) could lead to larger clinical improvement relative to amygdala-NF with neutral feedback interface (Neutral-NF); and (c) Demonstrate transferability of amygdala-neuromodulation in the treatment group, relative to No-NF, by showing greater amygdala-BOLD downregulation as measured by fMRI.

**Fig. 2:**
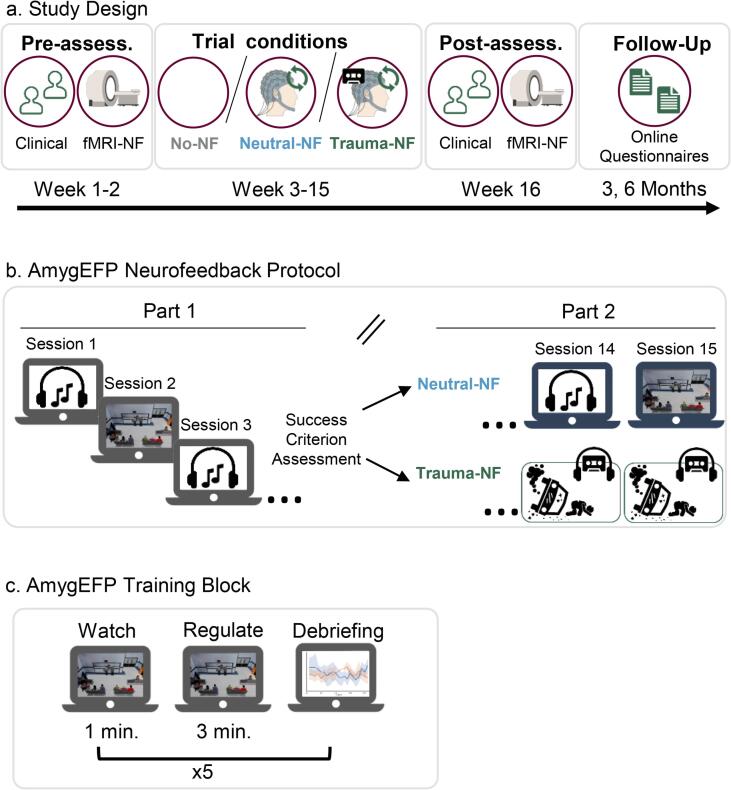
**Study Design and Neurofeedback Protocola. Study Design.** Clinical assessments, structural and fMRI-NF scans were performed at baseline (‘Pre’) and immediately after the completion of the intervention (‘Post’). Following baseline assessment patients were randomized either to the control group (No-NF group) or to the intervention groups (either Trauma-NF or Neutral-NF). Follow-up assessments were performed at 3 and 6 months following ‘Post’ using online questionnaires. **b. AmygEFP-NF Protocol.** The intervention phase included 15 training sessions, starting twice weekly for two weeks and then once a week (total of 13 weeks). The first 5 sessions were identical for both groups and employed two types of feedback interfaces in an interleaved manner: neutral auditory tune and audio-visual scenario. Patients assigned to the Neutral-NF group continued to train using these interfaces in an interleaved manner until the completion of 15 intervention sessions, while those in the Trauma-NF group who met the success criteria, moved on to the second phase of training with individually-tailored trauma-related feedback interface (see Fig. 1). **c. AmygEFP Training Block** (shown for a block with audio-visual scenario feedback). Each session (from all feedback types) consisted of 5 repetitions of 3 consecutive conditions: passive watch/listen baseline (1 min), active regulate (3 min) and a debriefing with a graphic feedback on the signal modulation time-course. During regulate participants were instructed to down-regulate the feedback stimuli by practicing self-generated mental strategies.

Fifty-nine adults meeting DSM-5 criteria for PTSD were randomized between Trauma-NF, Neutral-NF, or No-NF (Fig. 2). The intervention consisted of 15 AmygEFP-NF sessions. Prior to trauma narrative integration, patients performed NF guided by a non-trauma-related interface (interleaved audiovisual animated scenario (Cohen et al., 2016) and neutral auditory feedback (Keynan, 2016). In the Trauma-NF group, upon reaching a predetermined neuromodulation success criterion, NF training was further guided by a pre-recorded edited script of the personal traumatic experience including trauma hot-spots, thoughts, emotions, and physical sensations (Shalev et al., 1993, Rauch, 1996). Successful down-regulation of AmygEFP was reflected by the reduced sound volume of the traumatic script. PTSD symptoms were blindly assessed before and immediately after the NF training period as well as via self-report at 3- and 6-months follow-up. Lastly, to verify that patients learned amygdala down-regulation as previously shown in healthy participants (Keynan, 2016, Keynan, 2019), amygdala targeted fMRI-NF was performed by all groups before and after the NF training period.

We hypothesized that: (a) Both Trauma-NF and Neutral-NF would exhibit learned AmygEFP down-regulation; (b) Relative to No-NF, patients in the NF groups (Trauma- and Neutral-NF) would exhibit a larger reduction of PTSD severity following the intervention, maintained at 3- and 6-months follow-up; (c) Relative to Neutral-NF, Trauma-NF would result in a larger reduction of PTSD severity and (d) AmygEFP-NF would result in improved regulation of Amygdala-BOLD as indicated by fMRI-NF.

## 2 Method

### 2.1 Ethics statement

The authors assert that all procedures contributing to this work comply with the ethical standards of the relevant national and institutional committees on human experimentation and with the Helsinki Declaration of 1975, as revised in 2008. All procedures involving human subjects/patients were approved by Tel-Aviv Sourasky Medical Center Institutional Review Board and registered at ClinicalTrials.gov (NCT02544971).

### 2.2 Participants and recruitment

Starting April 2016, participants exhibiting post-traumatic stress symptoms (aged 18–65), were recruited through mental health clinics and social media advertisements. The screening process included a clinical assessment by a trained psychologist (Structured Clinical Interview for DSM-IV axis I disorders (SCID) and Clinician-Administered PTSD Scale (CAPS-5)). All patients gave written informed consent and received monetary compensation.

Exclusion criteria included current pregnancy, major medical or neurological disorders, psychosis, schizophrenia, and serious suicidal ideations. Participants who were currently in psychotherapy and/or were receiving pharmacological treatments were included in the study, on the condition that no change was made to their treatment plan in the last three months and in the time remaining to the completion of the study.

### 2.3 General procedure

Following screening, fifty-nine patients meeting CAPS-5 criteria for PTSD were randomized and forty patients completed the trial: Neutral-NF n = 14, Trauma-NF n = 13, No-NF n = 13 (CONSORT diagram in Supplementary Fig. S2). Fig. 2a outlines the general procedure of the trial. Outcome measurements were obtained Pre- and Post AmygEFP-NF intervention and included blinded clinical evaluation and amygdala fMRI-NF scans. Follow-up assessments were performed at 3- and 6 months following NF training using self-report questionnaires. Randomization took place following baseline assessments using an adaptive randomization protocol (South East Wales Trials Unit) minimizing differences in age (<or ≥ 40 years) and time since trauma (<or ≥ 5 years). Primary clinical outcomes included CAPS-5 interview and PTSD Checklist (PCL) (Weathers et al., 1993) for the Pre- and Post- intervention time points. Secondary outcomes included State-Trait Anxiety Inventory (STAI) (Spielberger, 2010), Beck Depression Inventory-II (BDI-II) (Beck et al., 1988), Toronto Alexithymia Scale (TAS-20) (Bagby et al., 1994), and Emotion Regulation Questionnaire (ERQ) (Garnefski and Kraaij, 2007). There were no differences between groups at baseline in any of the clinical or demographic measures (Supplementary Table S1). Baseline secondary clinical outcomes are presented in Supplementary Table S3. The study eventually included one patient undergoing psychotherapy during the duration of the study (randomized to No-NF group) and one patient receiving psychiatric medication (SSRI; randomized to Neutral-NF group). Their respective Z scores (depicted in Supplementary Table S4) show that their clinical improvement does not considerably vary from that of their group mean and thus they could not be considered outliers.

AmygEFP-NF included 15 sessions over 13 weeks (twice weekly for two weeks and then once a week). Each AmygEFP-NF session lasted approximately 40 min including preparation time and began with a 3-minute EEG resting-state recording. The first 5 sessions were identical for both treatment groups (Neutral-NF and Trauma-NF) and employed two types of feedback interfaces in an interleaved manner: a neutral musical excerpt and a multimodal animated scenario (Fig. S2b and Supplementary Material for details). Generally, in the auditory interface patients were instructed to lower the sound volume of a repetitive jazz music piece with no lyrics. In the animated scenario interface, amygdala down-regulation was reflected by lowering the unrest level of a virtual hospital waiting room indicated by the number of virtual characters aggregating in front of a receptionist and the loudness of their voices. Each AmygEFP-NF session in each of the interfaces (Auditory, Animated Scenario, and Trauma-NF) consisted of three consecutive conditions, each repeating 5 times: passive listening to or watching the interface (Watch, 1 min), downregulating AmygEFP (Regulate; 3 min) and debriefing by a graph and open questions about the successfully employed mental strategies (Fig. 2c). Instructions to patients were to freely use mental strategies, intentionally being unspecific, allowing individual adoption of most effective strategies (see Supplementary Material for elaboration on patient strategies).

AmygEFP signal down-regulation was assessed by calculating a personal NF success index for each subject in each session (i.e. average of 5 NF blocks minus the average of baseline blocks, divided by average baseline standard deviation) using the following formula:

$$\mathrm{NFsuccess}=\frac{\mathrm{mean}\;regulate-mean\;baseline}{\mathrm{SD}\;baseline}$$

The success index is a continuous measure that can range from positive (meaning up regulation) to negative (meaning down regulation). We consider any negative value of this index as successful down regulation as it is calculated as the delta between the average of the NF cycles and the average of the baseline cycles, divided by the standard deviation of the baseline. The baseline on auditory sessions was the initial rest period and in the multimodal animation scenario, the baseline was the active baseline block in each training cycle.

Patients in the Neutral-NF group continued to train using the auditory and animated scenario interfaces in an interleaved manner until the completion of 15 sessions. Patients in the Trauma-NF group, who met a pre-determined success criterion (see criterion definition below), moved on to the traumatic-script NF, in which AmygEFP down-regulation was reflected by a reduction in the sound volume of the traumatic memory auditory script. This was done in order to enable a gradual shift from neutral to the trauma-feedback interface and to ensure that regulation with the more challenging trauma feedback signal could be based on previously practiced regulation skills. The first trauma NF session included an interview about the traumatic event using common methodology (Shalev et al., 1993, Rauch, 1996) to produce a scripted detailed chain of events, including thoughts, feelings, sensations, and contextual information. The interview was edited and recorded as a three-minute audio segment (second-person male voice in present tense). Subsequently, patients who successfully down-regulated the AmygEFP signal during this session, with a neutral NF interface, went on to train with the trauma-narrative feedback interface in the remaining sessions.

***AmygEFP-NF success criterion***

The criterion was successful down-regulation (according to NF success index) of AmygEFP signal during training in three out of the last five sessions, or in four out of six total sessions. According to the treatment protocol, patients who do not succeed were to continue to train in the neutral context for the remaining sessions. In practice, only one participant (out of 13 that received the intervention in the Trauma-NF group) failed to reach criteria and continued to train with neutral feedback, while most participants made the criteria around session 8 (see Supplementary Fig. S6 for the distribution of session number in reaching trauma-exposure criteria).

***Neutral auditory NF***

The auditory sessions included five consecutive training blocks of three minutes each. The volume of sound could increase or decrease as a function of AmygEFP signal power (in units of 10 dB each). Patients were guided to reduce the auditory tone by using a variety of self-generated mental strategies. Each training block concluded with patient debriefing on the techniques they employed and a graphic display depicting their AmygEFP activity throughout the block (i.e. NF success).

**Multimodal animated scenario online calculation**: The scenario features the sound of chatter and commotion in a busy emergency room. The scenario can gradually change from a resting state (all the people are seated and the volume is low) to an agitated state (people coming up to the receptionist and protesting loudly) and back again. The overall unrest level of the room is determined by the AmygEFP signal power. The ratio between characters sitting down and protesting at the counter is considered to be a two-state Boltzmann distribution, whose evolution is driven by a ‘virtual temperature’ whose value is derived from the momentary value of the targeted signal power (AmygEFP). The scenario uses the probability (P-value) of a momentary signal value during regulate to be sampled under the previous attend distribution. This P-value is used to determine the probability of virtual characters to be moving in the virtual room, with the character distribution updated accordingly. A matching soundtrack recorded inside a real hospital complements the system output. Three alternative soundtracks with different agitation levels were produced and switched according to the signal value. During the attend condition, 75% of the characters congregate at the front desk while expressing their frustration through body and verbal language. The system is implemented using the Unreal Development Kit game engine, which controls relevant animations (walking, sitting, standing, and protesting), as well as their transitions for individual characters.

#### 2.3.1 EEG Data recording

EEG data were acquired using the V-Amp^TM^ EEG amplifier (Brain Products^TM^, Munich Germany) and the BrainCap™ electrode cap with sintered Ag/AgCI ring electrodes (Falk-Minow Services^TM^, Herrsching-Breitburnn, Germany). Electrodes were positioned according to the standard 10/20 system. The reference electrode was between Fz and Cz. Raw EEG signal was sampled at 250 Hz and recorded using Brain Vision Recorder™ software (Brain Products).

**The AmygEFP model**: The AmygEFP model was previously developed by our lab to enable the prediction of localized activity in the amygdala using EEG only (Meir-Hasson et al., 2016). This was done by applying machine learning algorithms on EEG data acquired simultaneously with fMRI. The procedure resulted in a *Time-Delay X Frequency X weight* coefficient matrix. EEG data recorded from electrode Pz at a given time-point are multiplied by the coefficient matrix to produce the predicted amygdala fMRI-BOLD activity. Keynan (2016) validated the reliability of the AmygEFP in predicting amygdala BOLD activity by conducting simultaneous EEG-fMRI recordings using a new sample not originally used to develop the model.

**On-line calculation of AmygEFP power**: Online EEG processing was conducted via the RecView software (Brain Products). RecView makes it possible to remove cardio-ballistic artifacts from the EEG data in real-time using a built-in automated implementation of the average artifact subtraction method (Allen et al., 1998). AmygEFP data were collected from electrode Pz. RecView™ was custom modified to enable export of the corrected EEG data in real-time through a TCP/IP socket. Preprocessing algorithm and signal calculation models were compiled from Matlab R2009b™ to Microsoft.NET™ in order to be executed within the Brain Vision RecView™ EEG Recorder system. Data were then transferred to a MATLAB.NET compiled DLL that calculated the value of the targeted signal power every 3 seconds. See Supplementary Material for fMRI data acquisition, fMRI-NF, and Statistical tools.

## 3 Results

**Baseline clinical characteristics**. To examine differences between groups at baseline a one-way ANOVA was conducted with Group (Trauma-NF, Neutral-NF, No-NF) as the independent variable. Analysis did not reveal differences between groups at baseline in any of the clinical measures: CAPS-5 (F_(2,35)_ = 1.22, p = .306, η_p_^2^ = 0.06), PCL (F_(2,35)_ = 1.13, p = .33, η_p_^2^ = 0.06), STAI (F_(2,34)_ = 1.12, p = .33, η_p_^2^ = 0.06), BDI-II (F_(2,34)_ = 0.76, p = .47, η_p_^2^ = 0.04), TAS-20 (F_(2,31)_ = 0.71, p = .49, η_p_^2^ = 0.04), ERQ reappraisal (F_(2,34)_ = 1.15, p = .32, η_p_^2^ = 0.06), ERQ suppression (F_(2,34)_ = 1.74, p = .19, η_p_^2^ = 0.09).

### 3.1 AmygEFP-NF learning

**Fig. 3:**
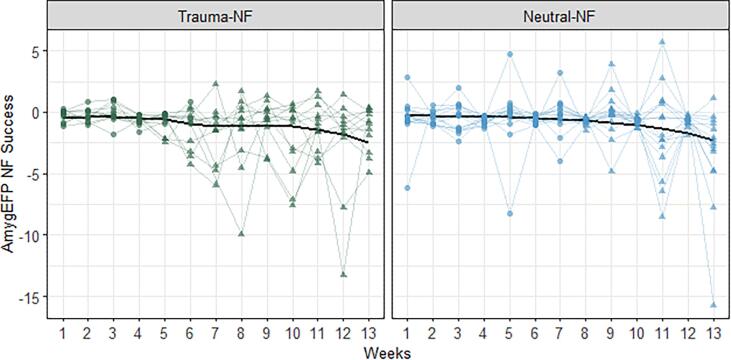
**NF Learning Effect**. Trauma-NF group (left panel) and Neutral-NF (right panel) individual AmygEFP-NF success (mean regulate minus mean baseline divided by baseline SD) as a function of intervention week. Training Stage is indicated by circle (Trauma-NF group weekly sessions with a neutral interface; Neutral-NF group weekly sessions 1–7) and triangle (Trauma-NF group sessions with trauma-narrative; Neutral-NF group sessions 8–13). Black line denotes predictions of time-point means made by the reported nested mixed-model, and smoothed via LOESS regression. Results show a significant reduction in AmygEFP signal over sessions and specifically greater down-regulation during the part 2 of the intervention.

Learned AmygEFP regulation was tested by a patient-repeated-measurements nested mixed-model analysis: Fixed effects of Group (Neutral-NF, Trauma-NF) and Time (13 weekly training sessions) and Group by Time interaction were fitted. Considering the nested structure of the study, we also included patient-specific random effects, including the random effects of Time nested within Group. Statistical significance was evaluated using likelihood ratio tests (LRT) and post-hoc pairwise comparisons. As hypothesized, both Neutral-NF and Trauma-NF showed successful AmygEFP down-regulation indicated by lower AmygEFP values during regulate relative to baseline (Negative intercept term significantly different from zero; Intercept = −0.549, LRT_(1, 271)_ = 36.01, p < .001). We further fitted a fixed effect for Training Stage (Part 1: Neutral-NF sessions 1–7, Trauma-NF sessions prior to exposure; Part 2: Neutral-NF sessions 8–13, Trauma-NF sessions including exposure). Results showed no fixed interaction with Time and Group factors (F_(12,259)_ = 1.67, p = .07) and no random interaction between these factors (χ^2^_(1)_ = 1.77, p = .18), yet demonstrated a significant fixed-main effect for change in AmygEFP signal over sessions (χ^2^_(1)_ = 4.91, p = .026). Post-hoc analysis further clarified that the main effect of Time was driven by greater AmygEFP down-regulation during the latter part of the intervention (p = .057, Fig. 3).

Further exploratory analysis showed no overall difference between Trauma-NF and Neutral-NF in AmygEFP down-regulation (χ^2^_(3)_ = 1.64, p = .65). Post-hoc comparison between groups conditioned on the effect of Training Stage, showed no difference between groups in either Part 1 (p = .48), or Part 2 (p = .48). Nevertheless, testing the effect of Training Stage within each group in order to assess change in modulation over time, revealed a significant improvement in AmygEFP-NF success at Part 2 of training for both groups (Neutral-NF p = .06, Trauma-NF p = .007). Assessing the monotonically decreasing curves from start to end, in each group, revealed that the Trauma-NF showed a numerically steeper learning slope, compared to Neutral-NF, however, the differences were not statistically significant (t_(24)_ = 0.49, p = .62).

### 3.2 Clinical outcome

A one-way ANCOVA was conducted to compare the clinical improvement (according to CAPS-5 and PCL) between groups (Neutral-NF, Trauma-NF, and No-NF) following AmygEFP-NF, while controlling for initial symptom severity (by entering either CAPS-5 or PCL as covariates). One participant was removed from this analysis for CAPS-5 and another for PCL, due to missing data at the Post-intervention timepoint, resulting in n = 38 and n = 37, respectively. Levene’s test and normality checks were carried out and the assumptions were met.

**Fig. 4:**
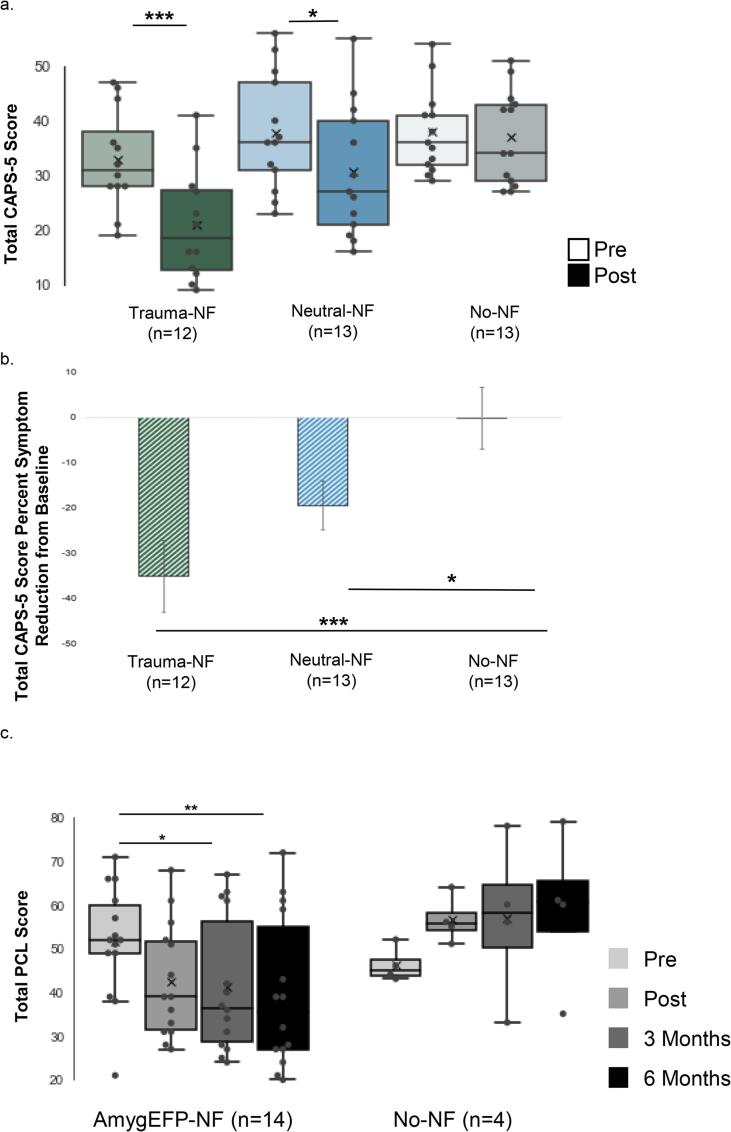
**Clinical Outcome Measures. a. Total CAPS-5** (Clinician Administered PTSD Scale) score reflecting the severity of PTSD symptoms at Pre and Post assessments for Trauma-NF, Neutral-NF and No-NF groups. Box represents first and third quartiles; the line represents the median while “x” represents the mean; whiskers depict minimum and maximum outside the first and third quartiles. Results demonstrate a significant reduction of CAPS-5 score following the intervention in the Neutral- and Trauma-NF groups, but not in the No-NF control group. At Post there was greater improvement in Trauma-NF compared to No-NF group and Neutral-NF group, while the difference between Neutral-NF and No-NF was not significant. **b. Total CAPS-5 Score Percent Symptom Reduction from Pre to Post.** Results demonstrate a significant difference between groups in percent of total symptom reduction according to CAPS-5. In comparison to No-NF group which showed no improvement, Trauma-NF showed the largest decrease, followed by Neutral-NF group. **c. Total PCL throughout the study:** Pre, Post, 3 and 6 months following the intervention. Results show a decrease in subjective PTSD severity following the intervention in both treatment groups, compared to No-NF control, throughout the study. Post-hoc analyses revealed a significant PCL reduction from Pre to 3 months follow-up and from Pre to 6 months follow up in the treatment arm.

As hypothesized, CAPS-5 total score showed a significantly greater improvement for AmygEFP-NF groups compared to No-NF, with the greatest improvement for Trauma-NF (F_(2,34)_ = 6.21, p = .005, η_p_^2^ = 0.26; Fig. S4a; for CAPS-5 subscales see Supplementary Results). Post-hoc tests showed a significant difference in CAPS-5 improvement for Trauma-NF (p = .001) and a marginal difference for Neutral-NF (p = .08), relative to No-NF, and a marginal difference between Trauma-NF and Neutral-NF (p = .07). Furthermore, the percent of symptom reduction in total CAPS-5 score differed between groups (F_(2,34)_ = 7.29, p = .002, η_p_^2^ = 0.30; Fig. 4b). Relative to No-NF which showed no improvement (-0.23%), Trauma-NF showed the largest decrease in symptoms (-35.13%; p = .001), followed by Neutral-NF (-19.48%; p = .04); the difference between Neutral-NF and Trauma-NF was marginal (p = .07). Number-Needed-to-Treat (NNT) was calculated based on achieving loss of PTSD diagnosis according to CAPS-5 after NF; comparing both treatment groups to No-NF showed NNT = 3.9 [ARR 25.54%, 95% CI −6.92% 58%] (Neutral-NF vs. No-NF: NNT = 6.5 [ARR 15.38%, 95% CI –22.48% 53.25%]; Trauma-NF vs. No-NF: NNT = 2.7 [ARR 36.54% 95% CI 0.49% 72.59%]). Intention-To-Treat (ITT) analysis of CAPS-5 and PCL further supported these results (see Supplementary Results).

The response rate in follow-up assessments was moderate (61.2%). To avoid bias, the analysis included patients that completed the PCL at all time points (Pre, Post, 3, and 6-months follow-up; resulting in n = 14 for AmygEFP-NF and n = 4 for No-NF). Results showed a larger decrease in PTSD severity for AmygEFP-NF relative to No-NF (Time by Group interaction F_(1,15)_ = 6.83, p = .02, η_p_^2^ = 0.31). Post-hoc analyses revealed PCL reduction from Pre-intervention to 3 months follow-up (p = .007) and from Pre-intervention to 6 months follow-up (p = .007) only for AmygEFP-NF (Fig. 4c).

### 3.3 Amygdala neuromodulation transferability

**Fig. 5:**
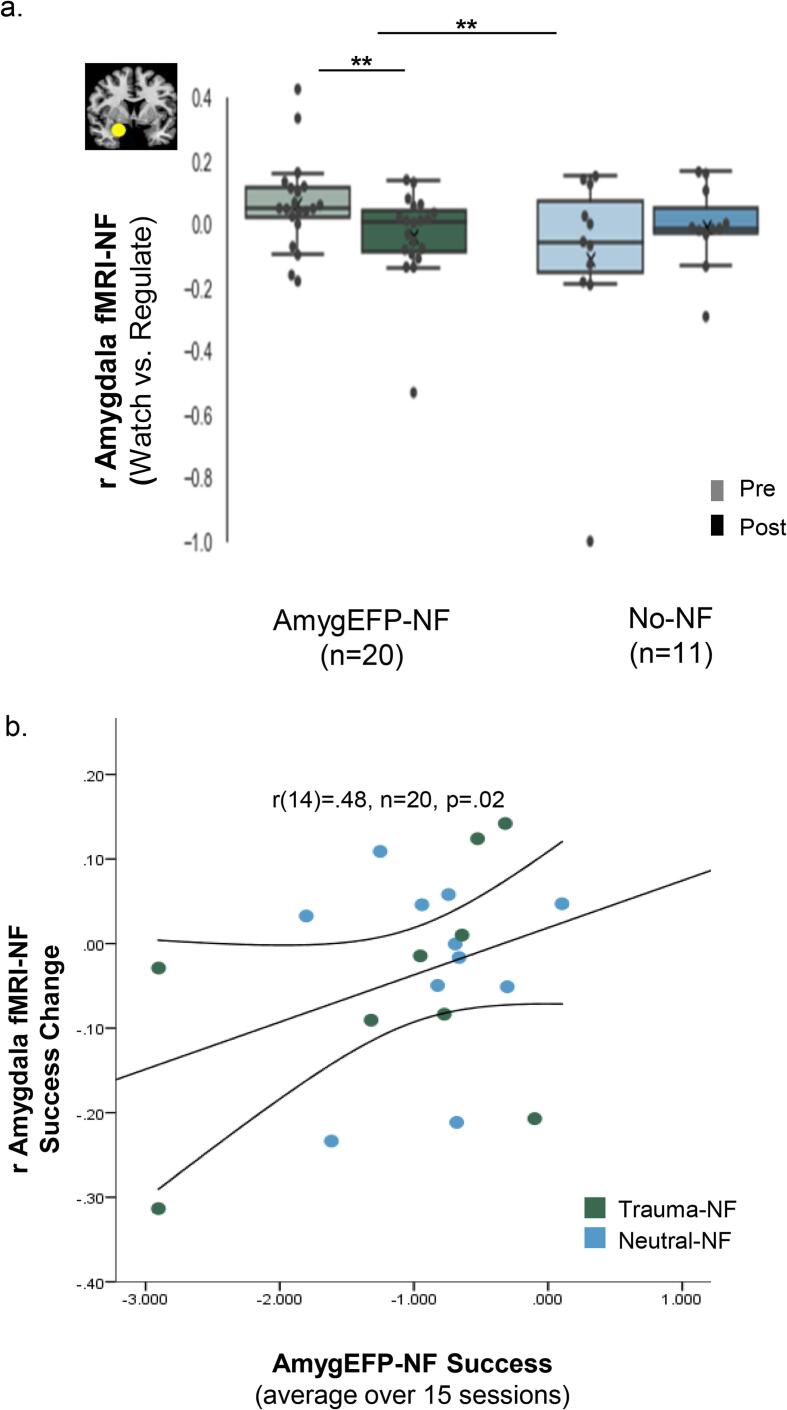
**a. Amygdala-BOLD NF before and after AmygEFP-NF training**. Average beta values obtained from the right amygdala used as the target for regulation during rtfMRI-NF. Regulation is depicted by the difference between regulate versus watch trials at Pre (the first cycle) and Post (averaged two cycles). Box represents first and third quartiles; the line represents the median while “x” represents the mean; whiskers depict minimum and maximum outside the first and third quartiles. Results showed that following AmygEFP-NF training, patients improved in their ability to down-regulate amygdala activity, compared to No-NF patients. **b. Association between Amygdala-BOLD-NF change and AmygEFP-NF training success.** Partial positive correlation (with mean CI) between AmygEFP success and right amygdala rtNF down-regulation change, controlling for age, time since trauma and gender. Trauma-NF and Neutral-NF indicated by green and blue dots, respectively.

As expected, fMRI-NF results showed greater amygdala-BOLD down-regulation following AmygEFP-NF relative to No-NF (Time by Group interaction F_(1,29)_ = 10.31, p = .004, η_p_^2^ = 0.26). Planned comparisons showed the desired effect for the NF groups (F = 10.85, p = .004), but not for No-NF (F = 2.41, p = .13) (Fig. 5a). Intriguingly, exploratory partial correlation analysis of the association between amygdala-BOLD down-regulation, clinical change, and AmygEFP learning showed a positive correlation (r_(14)_ = 0.48, n = 20, p = .02; Fig. 5b) between AmygEFP success (mean = -1.05, std = 0.78) and improved amygdala-BOLD down-regulation (mean = -0.4, std = 0.12). No correlation was found between AmygEFP-NF success and clinical improvement (CAPS-5: r_(14)_ = -0.09, p = .37; PCL: r_(14)_ = -0.02, p = .47; Supplementary Table S2).

## 4 Discussion

This study introduced a personalized and scalable process-based NF intervention for PTSD, aimed at amygdala down-regulation in PTSD patients. As hypothesized, AmygEFP-NF resulted in neuromodulation learning (i.e. down-regulation of the neural probe). As further hypothesized, both treatment groups improved clinically more than No-NF group, but AmygEFP-NF coupled with traumatic memory reinstatement resulted in the largest clinical effect. Lastly, as previously shown with healthy participants (Keynan, 2016, Keynan, 2019), AmygEFP-NF training resulted in improved amygdala-BOLD down-regulation following treatment, suggesting transferability of the skill to self-modulate the amygdala, as well as an association with NF learning.

### 4.1 NF learning

Overall, patients learned to down-regulate AmygEFP in both neutral and traumatic feedback interface contexts. The dynamic of learning across sessions showed that learning excelled during the second part of training. Interestingly, as can be seen in Fig. 3, the personalized trauma-narrative feedback did not interfere with learning and might have even accelerated it. Possibly, the gradual removal of an aversive cue (i.e. volume reduction of trauma-script) led to greater reward value and thus more rapid learning (Ferrucci et al., 2019). Another possibility is that the decrease (or increase) in the trauma-script volume affects the AmygEFP signal on its own, regardless of parallel reinforcement and regulation processes. This possibly confounding effect, should be further tested, systematically, in order to tease apart the effect of regulation with a trauma-script feedback interface, from that of the mere exposure to the decreasing trauma-script sound alone. To that end, we suggested that following this proof-of-concept study, a carefully controlled RCT will be conducted which will include a yoked-exposure-only group. Yet, it should be emphasized that since yoked-NF is an active control condition, it may introduce additional confounds on its own.

Additionally, the 15-session protocol was designed to match common psychotherapies; however, dose–effect should be further tested systematically.

### 4.2 Clinical effect

We found a relatively large clinical effect for AmygEFP-NF in chronic PTSD as indicated by total CAPS-5 score (Cohen's D 0.853; Hedges's g 0.591); Trauma-NF group showed a large effect size (Cohen's D 1.229; Hedges's g 0.828), and a medium effect size for Neutral-NF group (Cohen's D 0.636; Hedges's g 0.4306). Importantly, clinical change demonstrated here was driven by marked symptom reduction (according to CAPS-5) following treatment (Trauma-NF −35.13%, Neutral-NF −19.48%), compared to No-NF (-0.23%). Recovery according to CAPS-5 resulted in NNT = 3.9 and NNT = 2.4 for PCL. Overall, these clinical effects’ indicators are comparable with a recent meta-analysis that showed that prolonged exposure therapy (PE) in PTSD outperformed control conditions with a similarly large effect size (Hedges's g = 1.08, 95% CI 0.69 to 1.46) (Powers et al., 2010), and with reported NNT ≤ 4 for achieving loss of PTSD diagnosis following psychotherapy (Jonas, et al., 2013). One could argue that the large effect found for Trauma-NF could be due to the combination of NF and repeated exposure to traumatic content (Thibault, 2017). We consider, however, that this cannot entirely explain the extent of our results since Trauma-NF did not follow common practice of prolonged exposure therapy. In contrast, the exposure in Trauma-NF is of short duration (3 min), presenting only the gist of the traumatic memory in second-person and without home practice. Lastly, in contrast to psychotherapies, AmygEFP intervention does not require verbal interpersonal interaction, yet may show similar clinical benefits. All these differences may explain the relatively low dropouts of 10% in the Trauma-NF, and 15.7% in the Neutral-NF during the intervention phase (See Supplementary Fig. S2 for CONSORT diagram), whereas exposure-based treatments often result in high dropout rates of up to 40% (Imel et al., 2013).

Nevertheless, the clinical effect illustrated in this study is limited by the lack of a placebo arm. Previous works have emphasized the importance of including a Sham-NF group in order to control for unspecific effects of the training (i.e. expectation, motivation, etc.) (Schabus et al., 2017, Thibault, 2017). The current study design did not include a Sham-NF group, which prevented the assessment of a specific NF impact. Rather, it shows the ability of patients to learn downregulation of an electrical signal, even while listening to their traumatic script. Following this proof-of-concept study, a carefully controlled RCT is required to precisely evaluate the contribution of targeting the amygdala, over and above exposure, and possible placebo effects. To quantify the contribution of the exposure component, such an RCT could include a yoked-exposure-only group that will passively listen to the traumatic narrative (script in identical length and changing volume as in the Trauma-NF group), only without receiving contingent feedback of their brain signal. Additionally, including a placebo arm should take into consideration possible confounding effects of Sham-NF (Lubianiker et al., 2019). In general, active NF control conditions might introduce two types of confounds: (1) modulation of other processes that are not operated in the experimental intervention and (2) modulation of NF-general processes (i.e. Control, Reward, and Learning) that are essentially different from the experimental intervention. Using yoked-sham NF induces a lack of contingency between neural patterns and the feedback, which might lead to major differences in NF reward processes. That is, participants may deduce they are not receiving veritable feedback (Schabus et al., 2017) and thus may reduce their motivation, task engagement, and positive expectations in comparison to a genuine feedback group. Moreover, even when matching feedback variability between groups by ‘yoking’ in a double-blinded manner, there would still exist differences in NF learning, as no learning based on contingencies between feedback and neural patterns would occur. This confound relates to models of NF learning that stress the importance of associative (i.e., Hebbian) learning mechanisms that rely on contingencies between stimulus and response. To conclude, using Sham-NF as a control group has substantial disadvantages and the selection of a NF control arm is not a trivial task, and especially when dealing with patient populations. That is, NF sham-control is an active intervention and has ethical caveats. In any case, it is clear that large-scale NF studies with more control conditions are of utmost need for establishing NF as a treatment of choice in psychiatry. Considering all, we regard the current proof-of-concept study as a safety-feasibility trial, showing that PTSD patients are able to complete the procedure and benefit from it. The underlying mechanism of the observed clinical effect warrants further investigation.

In line with accumulating reports on the latent effect of NF (Rance et al., 2018, Goldway et al., 2019), 3- and 6-month follow-up assessments showed a continued reduction of PTSD severity as indicated by PCL. This finding should be interpreted with caution, due to the small sample size. One possible mechanism could be skill acquisition during NF that is subsequently practiced in daily life (intentionally or automatically) and thus improves over time. Another explanation could be that consolidation and reconsolidation processes, which are typical in NF learning, occur after training completion through synchronization of the targeted brain circuits.

Similar to Goldway et al. (Goldway et al., 2019) this study did not demonstrate a correlation between AmygEFP learning and clinical outcomes (see Supplementary Table S2). This is in opposition to our finding in a-priori healthy soldiers that underwent similar training for increasing stress resilience (Keynan, 2019). One possible explanation may be that clinical changes in patients may not be *linearly* related to the level of NF proficiency. Rather, skills are acquired and incorporated in behavioral repertoire (Kessler et al., 1995). Thus, the acquisition of the regulation skill and not the level of proficiency of that skill, could be the driving factor of clinical change. The small sample size of this proof-of-concept study limits the exploration of additional correlations (i.e., other than linear) and these should be examined in further larger studies.

### 4.3 Process-based NF

The current study demonstrates the potential of incorporating disorder-specific context in enhancing NF treatment efficacy (Lubianiker et al., 2019). Targeting of disorder-specific processes could be further explored by applying our trauma-script NF protocol during real-time fMRI-NF. Moreover, it could be argued that Since fMRI-NF tends to show a treatment effect with fewer sessions, than EEG-NF, symptom change following the initial baseline evaluation fMRI-NF sessions should be examined. It should be noted that in order to reduce any learning effects prior to AmygEFP-NF sessions, the initial fMRI-NF session conducted at baseline, was a short session which included only 2 cycles (of 60 s each) and was aimed only to test participants baseline regulation abilities (based on the protocol of (Keynan, 2016). In any case, examining patients' performance during the first fMRI-NF session, showed that learning did not occur at this stage since the difference between 'Baseline' and 'Regulate' conditions was non-significant (t = −0.65, p = .51). However, since symptom evaluation was performed prior to the fMRI-NF initial session, as part of the initial screening process (see Fig. 2a. General Procedure), it is still possible that symptoms were changed or affected by that session. Yet, as stated above this study did not find a correlation between fMRI-NF performance and clinical change (see Supplementary Table S2).

To note, patients who trained with AmygEFP NF using the trauma-narrative interface showed larger improvement relative to those trained with Neutral-NF, in several symptom clusters of CAPS-5 (intrusion, avoidance, and arousal, see Supplementary 2.1). Future studies, with a larger sample size, could explore specific process-related outcome measures (e.g. cognitive testing, emotional challenges, etc.) and pursue learning-specific manifestations as an additional indication for success, especially in patients. In addition, anxiety and depression symptoms were also improved over time in AmygEFP-NF groups and were even maintained during follow-up (see Supplementary Fig. S4b, c). PTSD has a high rate of comorbidity mainly with major depression, anxiety disorders, and substance use disorders (Kessler et al., 1995, Campbell, 2007). This highlights the importance of assessing comorbidity in PTSD but also supports our idea that the AmygEFP-NF probes an underlying mechanism of the disorder; emotion dysregulation, which could be relevant *trans*-diagnostically. Future studies could delineate clinical effects with regard to other processes underlying PTSD and comorbid pathologies; e.g. reward processing or cognitive control. Clearly, such characterization highlights the potential of personalization in the future implementation of NF in PTSD. Learning variability among individuals has been large, hence predicting treatment response using initial NF success (Alkoby et al., 2018, Paret et al., 2019, Kadosh and Staunton, 2019) or resting state EEG (Wu, 2020) could facilitate personalization and further enhance efficacy.

To conclude, the current study is a proof-of-concept for a scalable neurofeedback intervention that is informed by a neural mechanism underlying PTSD and its clinical impact. The integration of individually-tailored context in the NF training points to a new horizon for personalized psychiatric treatments that are brain-guided yet psychologically engaging and relevant.

#### CRediT authorship contribution statement

**Tom Fruchtman-Steinbok:** Conceptualization, Methodology, Software, Validation, Formal analysis, Investigation, Data curation, Writing – original draft, Writing – review & editing, Visualization, Project administration. **Jackob N. Keynan:** Validation, Writing – review & editing. **Avihay Cohen:** Methodology, Software, Validation, Formal analysis, Data curation. **Iman Jaljuli:** Software, Formal analysis, Visualization. **Shiri Mermelstein:** Investigation, Project administration, Data curation. **Gadi Drori:** Investigation. **Efrat Routledge:** Investigation, Project administration. **Michael Krasnoshtein:** Methodology, Resources. **Rebecca Playle:** Methodology, Software, Formal analysis, Data curation. **David E.J. Linden:** Writing – review & editing, Supervision, Funding acquisition. **Talma Hendler:** Conceptualization, Methodology, Writing – review & editing, Supervision, Funding acquisition.

## Declaration of Competing Interest

The authors declare the following financial interests/personal relationships which may be considered as potential competing interests: T.H is the inventor of related patent applications entitled “Method and system for use in monitoring neural activity in a subject's brain” (US20140148657 A1, WO2012104853 A3, EP2670299 A2). T.H and J.N.K are inventors of related patent applications entitled “Resilience training” (US201862767650P, US62/767,650, WO2020100144A1). This does not alter the authors' adherence to journal policies. All other authors declare no financial conflict of interest.
