# Supplementary material for: Amygdala electrical-finger-print (AmygEFP) NeuroFeedback guided by individually-tailored Trauma script for post-traumatic stress disorder: Proof-of-concept
Source: Neuroimage Clin. 2021 Oct 15;32:102859. doi: 10.1016/j.nicl.2021.102859 (PMC8551212; doi:10.1016/j.nicl.2021.102859)
Supplement: Supplementary data 1 [file mmc1.docx]

**Supplementary Material:**

**Amygdala Electrical-Finger-Print (AmygEFP) NeuroFeedback Guided by Individually-tailored Trauma Script for Post-Traumatic Stress Disorder: Proof-of-Concept**

Tom Fruchtman-Steinbok^1,2^, Jackob N. Keynan^1,7^, Avihay Cohen^1,2^, Iman Jaljuli^5^, Shiri Mermelstein^1^, Gadi Drori^1,3^, Efrat Routledge^1^, Michael Krasnoshtein^6^, Rebecca Playle^8^, David E.J. Linden^9^, Talma Hendler^1,2,3,4*^

^1^Sagol Brain Institute, Tel-Aviv Medical Center, Tel-Aviv, Israel

^2^School of Psychological Sciences, Gershon H. Gordon Faculty of Social Sciences, Tel-Aviv University, Tel-Aviv, Israel

^3^Sagol School of Neuroscience, Tel-Aviv University, Tel-Aviv, Israel

^4^Sackler Faculty of Medicine, Tel-Aviv University, Tel-Aviv, Israel

^5^Department of Statistics and Operations Research, School of Mathematical Sciences, Tel-Aviv University, Tel-Aviv, Israel

^6^Psychiatric Department, Tel-Aviv Sourasky Medical Center, Tel-Aviv, Israel

^7^Department of Psychiatry & Behavioral Science, Stanford University School of Medicine, Stanford, CA, USA

^8^Center for Trials Research, College of Biomedical & Life Sciences, Cardiff University, Cardiff, UK

^9^Division of Psychological Medicine and Clinical Neurosciences, School of Medicine, Cardiff University, Cardiff, UK

*Corresponding author: [hendlert@gmail.com](mailto:hendlert@gmail.com)

**Method**

**fMRI Data Acquisition and Processing:** To allow high-resolution structural images, a T1-weighted three-dimensional (3D) sagittal MPRAGE pulse sequence (repetition time/echo time = 1,860/2.74 ms, flip angle = 8°, pixel size = 1 × 1 mm, field of view = 256 × 256 mm) was used. Functional whole-brain scans were performed in an interleaved top-to-bottom order, using a T2*-weighted gradient echo planar imaging pulse sequence (repetition time/echo time = 2,500/30 ms, flip angle = 82°, pixel size = 2.3 mm, field of view = 220 × 220 mm, slice thickness = 3 mm, 42 slices per volume). Slice scan time correction was performed using cubic-spline interpolation. Head motions were corrected by rigid body transformations, using three translations and three rotation parameters, and the middle image served as a reference volume. Trilinear interpolation was applied to detect head motions, and sinc interpolation was used to correct them. The temporal smoothing process included linear trend removal and use of a high-pass filter of 1/128 Hz. Functional maps were manually co-registered to corresponding structural maps and, together, they were incorporated into 3D data sets through trilinear interpolation. The complete data set was transformed into Talairach space and spatially smoothed with an isotropic 6-mm full width at half-maximum Gaussian kernel. The model included six regressors for each condition in each cycle (Baseline, NF and Washout) in PRE and POST separately. Regressors were convolved with a canonical hemodynamic response function. Additional nuisance regressors included the head-movement realignment parameters.

**Real-time fMRI-NF:** Patients completed one session of amygdala-fMRI-NF before and after AmygEFP-NF training (Pre and Post, Figure 2a). Structural and functional MRI scans were performed in a 3.0T Siemens MRI system (MAGNETOM Prisma) using a 20-channel head coil. Preprocessing and statistical analysis were performed using BrainVoyager QX version 2.8 (Brain Innovation). The probing of amygdala-BOLD for NF was based on a 6-mm sphere in Talairach space in the right amygdala (coordinates, 20, -5, -14) in correspondence to the Amygdala BOLD used as a Predictor for the AmygEFP model. The reinforcement feedback for successful down-regulation of the amygdala BOLD was the change in an animated visual interface different from the one used for the AmygEFP-NF training. The change in amygdala activity during regulate block was represented by the speed of a skater figure^23,24^. Patients were instructed to lower the skater's speed. During the first session they were instructed to use any mental strategy they see fit, while during the second session they were encouraged to use techniques they found successful during the training phase (for patients in the treatment arm). Decreased speed corresponded with decreased amygdala BOLD activity during NF relative to watch condition (see Supplementary Figure 3).

**fMRI-NF paradigm online calculation** – Momentary beta weights of the predefined amygdala ROI (averaged across all voxels of the ROI) were extracted online using Turbo Brainvoyager 3.0 (Brain Innovation). The beta weights were then transferred to MATLAB^TM^. For each TR, right amygdala activity was first converted to standardized score:

$$zBold\left( t \right)=\frac{B\left( t \right)- \mu\left( B_{BL} \right)}{\sigma\left( B_{BL} \right)}$$

Where B(t) is the right amygdala BOLD activity value at time point *t*, $\mu\left( B_{BL} \right)$ is the mean BOLD value during the Previous 'Active baseline'. Values varied from -4 std. below baseline average to 4 std. above average. Next, each value was transferred into a speed scale using the following formula:

$$Speed\left( t \right)=60+15*zBold\left( t \right)$$

This equation results in the fact that the right amygdala BOLD value that equals the Previous 'Active baseline' average value, is set to 60km/h and each increase or decrease of 1 std. results in a 15km/h change. The -4 to 4 limit translates to a possible speed range of 0-120km/h. Moreover, in order to make the NF paradigm more interactive, flexible for modulation and challenging, an updating principle was incorporated. During NF block, represented values were limited by an interval of 4 std., resulting in a 60km/h interval in speed scale. In the first block of the first run, the presented interval was symmetric around the average speed (60km/h): -2 to 2 (30km/h to 90km/h). In each consecutive run, the interval was updated based on the patients' success in the previous run, according to the following steps:

1. During the first cycle the interval in the NF screen is set to -2 to 2.
2. When the NF screen terminates a success index of the current block is calculated thus (n indicating the cycle's ordinal number):

$$Success_{Index\left( n \right)}=\frac{\mu\left( B_{NF\left( n \right)} \right)}{pooled\left( \sigma\left( B_{BL\left( n \right)} \right)+ \sigma\left( B_{NF\left( n \right)} \right) \right)}$$

By dividing the mean NF standardized value with the pooled average, we introduce the index not only with the subject's ability to successfully regulate his or her right amygdala activity during the NF block, but also his or her ability to maintain it as constant as possible during the 'Active baseline' block.

1. The interval [lower limit upper limit] for the next cycle (n+1) is updated to be [Lower lim = (success_index(n) - learning_rate) Upper lim = (success_index(n) - learning_rate + 4)] The learning rate parameter is preset to: learning rate = 1.

This process is aimed at enabling significant regulation in one run to result in further regulation in the following run. This makes the NF paradigm more challenging and dynamic and pushes towards maximizing regulation.

**Statistics:** R version 3.6 (R Core Team, 2019, Vienna, Austria; lme4 package), SPSS version 20 (IBM Corp., 2011, Armonk, N.Y.) and STATISTICA version 10 (StatSoft, Inc., 2011) were used for statistical analysis. Multiple comparisons correction was performed using FDR (≤.05) for each hypothesis.

**Results**

**AmygEFP-NF strategies**

Patients were instructed to freely use mental strategies, intentionally being unspecific, allowing individual adoption of most effective strategies. Patients in the Trauma-NF group reported experiencing intruding images of the traumatic event in the trauma sessions. After a few sessions some learned to anticipate peak moments in the script, which were accompanied by heightened arousal (elevation of heartbeat, sweaty hands, dry mouth). Some Trauma-NF patients reported re-living parts of the trauma, while others would have preferred the induction to be more emotionally activating by including sounds of explosions and such. The strategies patients in both AmygEFP groups reported could be classified into several categories (sometimes several strategies were used simultaneously):

1. **Engagement with the feedback interface** – in sessions with neutral context this included telling the people in the reception area (of the audiovisual interface) to sit down or imagine their lives; focusing on one part of the interface for example the soft drinks machine or the bass sounds in the auditory feedback; imagining yourself as one of the musicians playing in the auditory interface. In the trauma sessions this included listening; focusing on details and keeping attention on the script; processing emotions and thoughts that come up while listening such as self-blame and anger; thinking about more positive interpretations and outcomes of the traumatic event or replacing negative valanced words with positive ones, practicing empathy to oneself while listening.

**2. Mental Imagery** – imagining diving deeper into the ocean, waves advancing and retreating on a beach, going down in an elevator, riding a bike, sailing, cooking, turning down a volume dial, imagining giving a lecture about the traumatic event, driving down the coast, imagining relaxing colors or a growing white light orb.

**3. Mental rehearsal** - repeating a mantra, going over a schedule or to-do list, arithmetic calculations or counting.

4. Focus on breathing, practice body scanning.

5. **Recall** - thinking about loved ones or pets; scenes or places with positive emotional valence, or joyful events that occurred after the traumatic event.

**AmygEFP-NF Clinical Efficacy**

**PCL analysis** revealed, as hypothesized, that patients in treatment groups improved more than the No-NF group (Time by Group interaction F_(2,34)_=4.403, p=.02, η_p_^2^=0.20; Time main effect F_(1,34)_=6.14, p=.02, η_p_^2^=0.15; see Figure 3a.). Planned contrasts revealed a PCL reduction from Pre to Post in the Neutral-NF group (Δ=-9.07; F=9.13, p=.01) and in the Trauma-NF group (Δ=-7.16; F=5.25, p=.02), but not in the No-NF group (Δ=-2.84; F=0.92, p=.36). NNT for the change in PCL score, comparing treatment groups to No-NF was 2.4.

We additionally explored the clinical effect with respect to type of feedback (trauma vs. neutral) by examining changes in CAPS-5 subscale in the treatment groups (i.e. Intrusion, Avoidance, Alterations in Cognition and Mood, Arousal). Two-way repeated measures ANOVAs were performed for each CAPS-5 subscale with Time (Pre, Post) as within- and Group (Neutral-NF and Trauma-NF) as between-subject independent variables. All scales demonstrated a similar improvement over time with no Time by Group interaction (Time main effect: Avoidance F_(1,23)_=15.75, p=.0006, ηp2=.406; Intrusion F_(1,23)_=9.73, p=.004, ηp2=.29; Alteration in Cognition and Mood F_(1,23)_=15.43, p=.0004, ηp2=.41; Arousal F_(1,23)_=16.54, p=.004, ηp2=.41, see Supplementary Figure 5). Although no significant interactions were found, in further Post-hoc tests it was evident that for all subscales there was a smaller decrease in symptoms for the Neutral-NF relative to the Trauma-NF group (Time simple main effect: Intrusion: Trauma-NF Δ=-1.5, F=6.34, p=.01; Neutral-NF Δ=-1.07, F=3.54, p=.072; Avoidance: Trauma-NF Δ=-2.75, F=12.46, p=.001; Neutral-NF Δ=-1.54, F=4.22, p=.051; Alteration in Cognition and Mood: Trauma-NF Δ=-4.25, F=10.204, p=.004; Neutral-NF Δ=-3.23, F=6.38, p=.01; Arousal: Trauma-NF Δ=-3.42, F=16.11, p=.0005; Neutral-NF Δ=-1.39, F=2.86, p=.103).

**Intention-To-Treat Analysis:** Expanding upon the evaluation of clinical change following AmygEFP intervention, we used intention-to-treat analysis in order to evaluate change in total CAPS-5 and PCL scores over time; including all randomized patients who completed at least one NF session. To that end, we applied mixed-models analysis. Analysis of total CAPS-5 score had fixed effects for Group (Neutral-NF, Trauma-NF and No-NF) and Time (Pre and Post intervention) as well as their interaction. We also included a random effect for the different patients nested within a binary variable of drop-out status. Analysis of total PCL score had similar fixed effects of Group (Neutral-NF, Trauma-NF and No-NF) and Time (Pre, Post, 3 months and 6 months following the intervention) as well as their interaction. We also included a random effect for the different patients. Evaluation of statistical significance was performed using ANOVA test.

CAPS-5 results demonstrated a significant effect for Time (F_(1,38)_=18.80, p=.00009, pFDR=.0005) and for Group (F_(2,48)_=7.21, p=.001, pFDR=.003), and a non-significant trend for Time by Group interaction (F_(2,42)_=2.78, p=.073, pFDR=.087). Post-hoc analysis using independent sample t-tests showed a significant improvement in severity following the intervention the Trauma-NF group (t_(26)_=2.83, p=.009, pFDR=.018), Neutral-NF group showed a marginal effect (t_(28)_=1.88, p=.069, pFDR=.087) and No-NF group showed no significant change (t_(26)_=.76, p=.45, pFDR=.45).

PCL results showed a significant effect for Time (F_(3,81)_= 5.04, p=.002, pFDR=.004) and Group (F_(2,47)_=5.19, p=.009, pFDR=.016) as well as their interaction (F_(6,81)_=3.75, p=.002, pFDR=.004). Post-hoc analysis using independent sample t-tests showed that the AmygEFP-NF groups showed a marginally significant reduction in PCL scores from Pre to Post (t_58_=1.92, p=.059, pFDR=.088), this reduction was also maintained at 3 months follow-up (t_48_=3.65, p=.001, pFDR=.004) and also at 6 months follow-up (t_47_=3.82, p=.0003, pFDR=.002). This reduction in PCL scores over time was not observed in the No-NF group (Pre to Post t_25_=-.16, p=.98, pFDR=.98; Pre to 3 months follow-up t_18_=.24, p=.807, pFDR=.308; Pre to 6 months follow-up t_19_=.15, p=.87, pFDR=.97).

**Secondary Clinical Outcomes**

We employed a set of secondary clinical assessments based on self-rated questionnaires: alexithymia; TAS-20 (n=33), depression; BDI-II (n=35), anxiety; STAI (n=35) and emotion regulation; ERQ (n=35). Two-way repeated measures ANOVAs were conducted with Time (Pre, Post) as within- and Group (Neutral-NF, Trauma-NF and No-NF) as between-subject independent variables.

Results showed improvement in BDI-II scores over time for the treatment arm, compared to No-NF group (Time by Group interaction F_(2,32)_=3.48, p=.04, ηp2=.17; Time main effect F_(1,32)_=7.28, p=.01, ηp2=.18, Figure 3b.). Post-hoc analysis revealed reduced depression symptoms in the Neutral-NF group (Time simple main effect; F=11.14, p=.002; Δ=-11.92), and marginal reduction for Trauma-NF group (F=3.47, p=.07; Δ=-5.16), but no change in the No-NF group (F=.19, p=.66; Δ=-4.08). Analysis of change in the STAI scale, showed reduced anxiety symptoms over time in the treatment arm but not in the control group (marginal Time by Group interaction F_(2,32)_=3.28, p=.0504, ηp2=.17, Time main effect F_(1,32)_=4.69, p=.03, ηp2=.12, Figure 3c.). Post-hoc analysis revealed reduced anxiety symptoms in the Neutral-NF group (Time simple main effect; F=6.8, p=.01; Δ=-9.91), and in the Trauma-NF group (F=4.24, p=.04; Δ=-7.83), but no change in the No-NF group (F=.67, p=.41; Δ=3.27). Analysis of change in alexithymia and emotion regulation did not reveal differences between treatment and No-NF arms: TAS-20 showed no Time by Group interaction (F_(2,30)_=.66, p=.52, η_p_^2^=0.04); ERQ score was divided into two scales: reappraisal and suppression, a significant Time by Group interaction was found for reappraisal (F_(2,32)_=5.03, p=.01, η_p_^2^=.23) but not for suppression (F_(2,32)_=.11, p=.89, η_p_^2^=.007).

**Amygdala fMRI NF:** Amygdala activity was compared between patients in AmygEFP treatment arm and No-NF arm via a random-effects general linear model for amygdala signal change (i.e. regulate vs. watch). A 2-way repeated measures ANOVA was conducted with right amygdala beta values as a dependent variable, and Group (AmygEFP-NF, No-NF) and Time (Pre, Post) as independent variables. Six patients were originally not included in the fMRI testing due to MR incompatibility, three additional patients were excluded due to missing data at one time point and two additional patients were excluded due to excessive head motion, resulting in n=20 in AmygEFP-NF group and n=11 in No-NF group, for the fMRI-NF analysis.
