# Supplementary material for: Amygdala electrical-finger-print (AmygEFP) NeuroFeedback guided by individually-tailored Trauma script for post-traumatic stress disorder: Proof-of-concept
Source: Neuroimage Clin. 2021 Oct 15;32:102859. doi: 10.1016/j.nicl.2021.102859 (PMC8551212; doi:10.1016/j.nicl.2021.102859)
Supplement: Supplementary data 2 [file mmc2.pptx]

## Slide 1
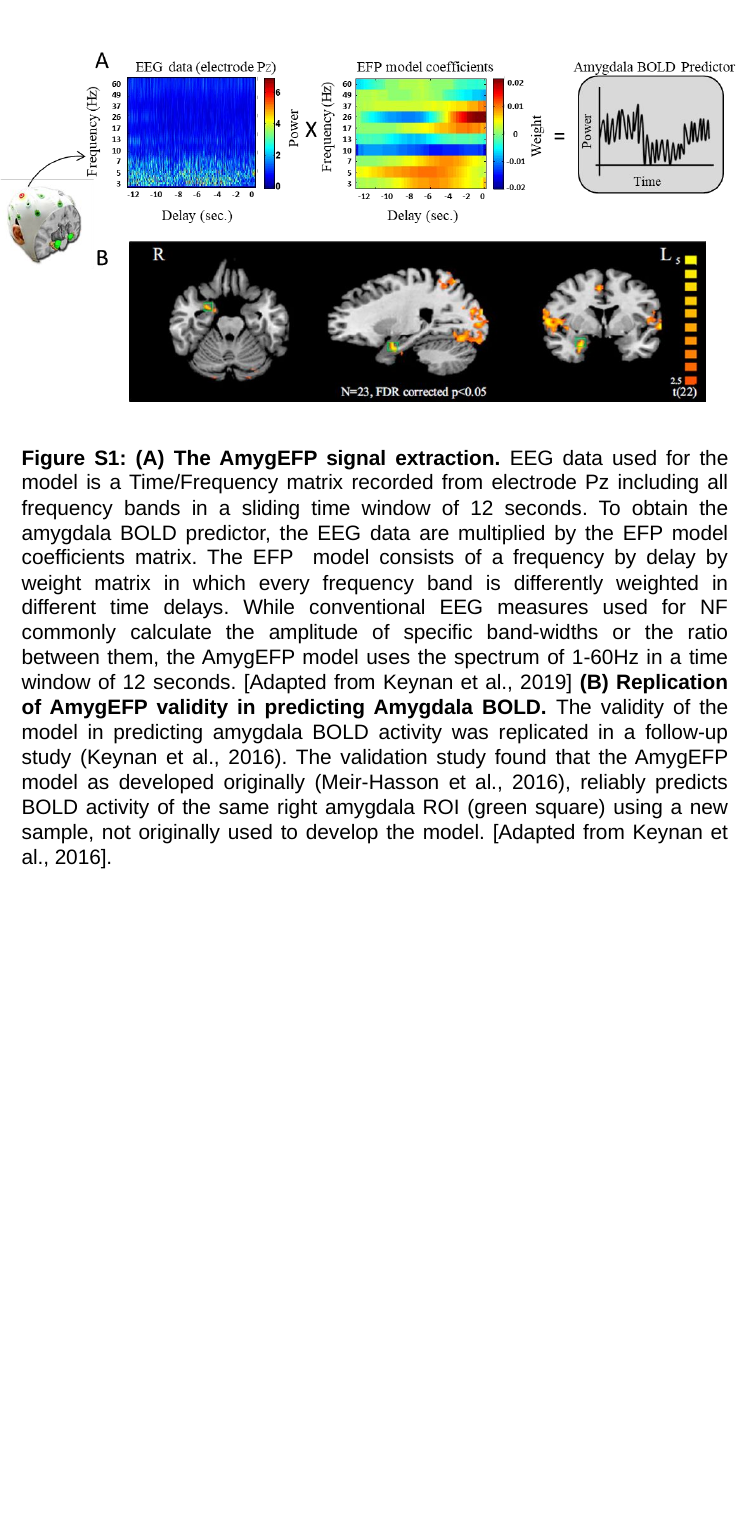

Figure S1: (A) The AmygEFP signal extraction. EEG data used for the model is a Time/Frequency matrix recorded from electrode Pz including all frequency bands in a sliding time window of 12 seconds. To obtain the amygdala BOLD predictor, the EEG data are multiplied by the EFP model coefficients matrix. The EFP model consists of a frequency by delay by weight matrix in which every frequency band is differently weighted in different time delays. While conventional EEG measures used for NF commonly calculate the amplitude of specific band-widths or the ratio between them, the AmygEFP model uses the spectrum of 1-60Hz in a time window of 12 seconds. [Adapted from Keynan et al., 2019] (B) Replication of AmygEFP validity in predicting Amygdala BOLD. The validity of the model in predicting amygdala BOLD activity was replicated in a follow-up study (Keynan et al., 2016). The validation study found that the AmygEFP model as developed originally (Meir-Hasson et al., 2016), reliably predicts BOLD activity of the same right amygdala ROI (green square) using a new sample, not originally used to develop the model. [Adapted from Keynan et al., 2016].
