# Supplementary material for: Amygdala electrical-finger-print (AmygEFP) NeuroFeedback guided by individually-tailored Trauma script for post-traumatic stress disorder: Proof-of-concept
Source: Neuroimage Clin. 2021 Oct 15;32:102859. doi: 10.1016/j.nicl.2021.102859 (PMC8551212; doi:10.1016/j.nicl.2021.102859)
Supplement: Supplementary data 3 [file mmc3.pptx]

## Slide 1
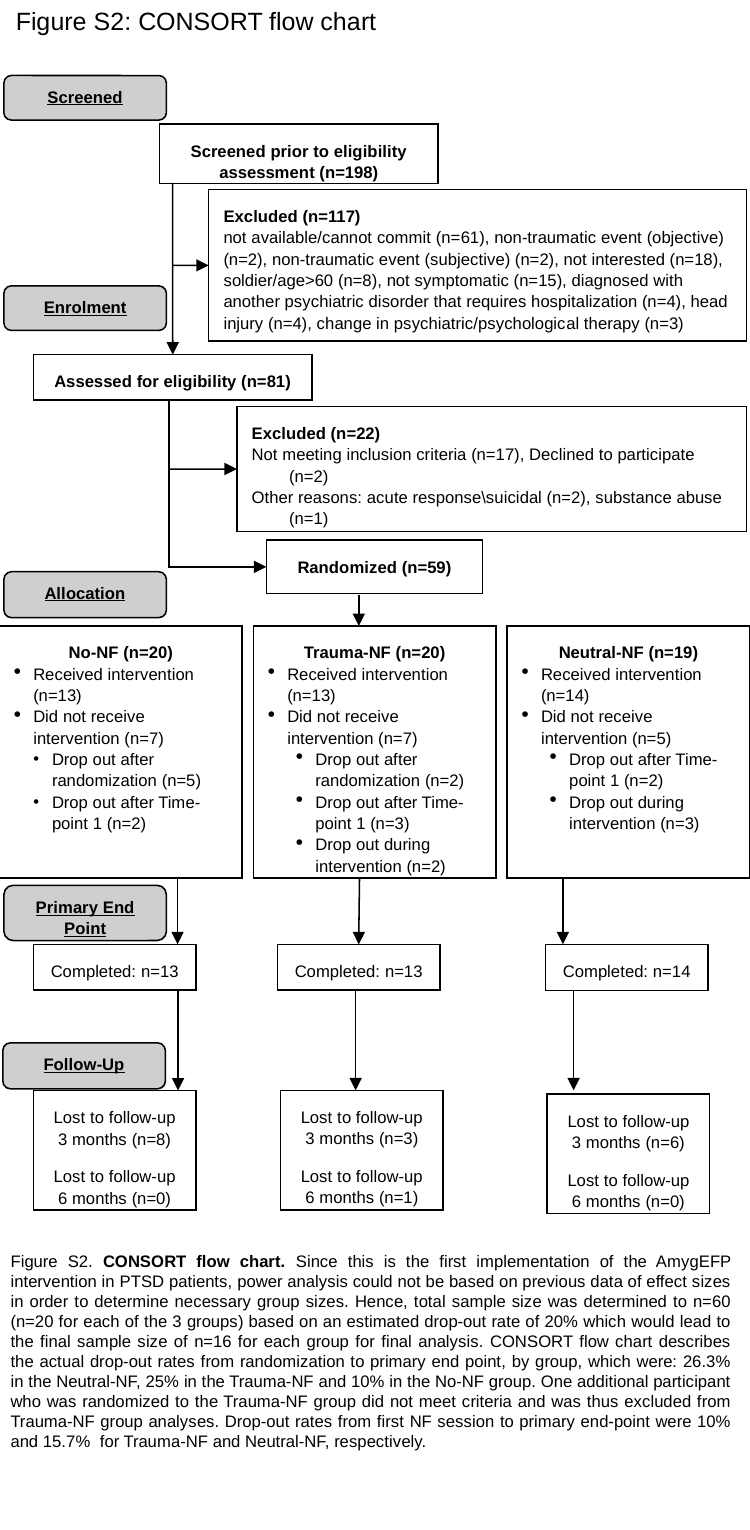

Figure S2: CONSORT flow chart
Screened
Screened prior to eligibility assessment (n=198)
Excluded (n=117)
not available/cannot commit (n=61), non-traumatic event (objective) (n=2), non-traumatic event (subjective) (n=2), not interested (n=18), soldier/age>60 (n=8), not symptomatic (n=15), diagnosed with another psychiatric disorder that requires hospitalization (n=4), head injury (n=4), change in psychiatric/psychological therapy (n=3)
Enrolment
Assessed for eligibility (n=81)
Excluded (n=22)
Not meeting inclusion criteria (n=17), Declined to participate (n=2)
Other reasons: acute response\suicidal (n=2), substance abuse (n=1)
Randomized (n=59)
Allocation
No-NF (n=20)
Received intervention (n=13)
Did not receive intervention (n=7)
Drop out after randomization (n=5)
Drop out after Time-point 1 (n=2)
Trauma-NF (n=20)
Received intervention (n=13)
Did not receive intervention (n=7)
Drop out after randomization (n=2)
Drop out after Time-point 1 (n=3)
Drop out during intervention (n=2)
Neutral-NF (n=19)
Received intervention (n=14)
Did not receive intervention (n=5)
Drop out after Time-point 1 (n=2)
Drop out during intervention (n=3)
Primary End Point
Completed: n=13
Completed: n=13
Completed: n=14
Follow-Up
Lost to follow-up 3 months (n=3)
Lost to follow-up 6 months (n=1)
Lost to follow-up 3 months (n=8)
Lost to follow-up 6 months (n=0)
Lost to follow-up 3 months (n=6)
Lost to follow-up 6 months (n=0)
Figure S2. CONSORT flow chart. Since this is the first implementation of the AmygEFP intervention in PTSD patients, power analysis could not be based on previous data of effect sizes in order to determine necessary group sizes. Hence, total sample size was determined to n=60 (n=20 for each of the 3 groups) based on an estimated drop-out rate of 20% which would lead to the final sample size of n=16 for each group for final analysis. CONSORT flow chart describes the actual drop-out rates from randomization to primary end point, by group, which were: 26.3% in the Neutral-NF, 25% in the Trauma-NF and 10% in the No-NF group. One additional participant who was randomized to the Trauma-NF group did not meet criteria and was thus excluded from Trauma-NF group analyses. Drop-out rates from first NF session to primary end-point were 10% and 15.7% for Trauma-NF and Neutral-NF, respectively.
