# Supplementary material for: Amygdala electrical-finger-print (AmygEFP) NeuroFeedback guided by individually-tailored Trauma script for post-traumatic stress disorder: Proof-of-concept
Source: Neuroimage Clin. 2021 Oct 15;32:102859. doi: 10.1016/j.nicl.2021.102859 (PMC8551212; doi:10.1016/j.nicl.2021.102859)
Supplement: Supplementary data 4 [file mmc4.pptx]

## Slide 1
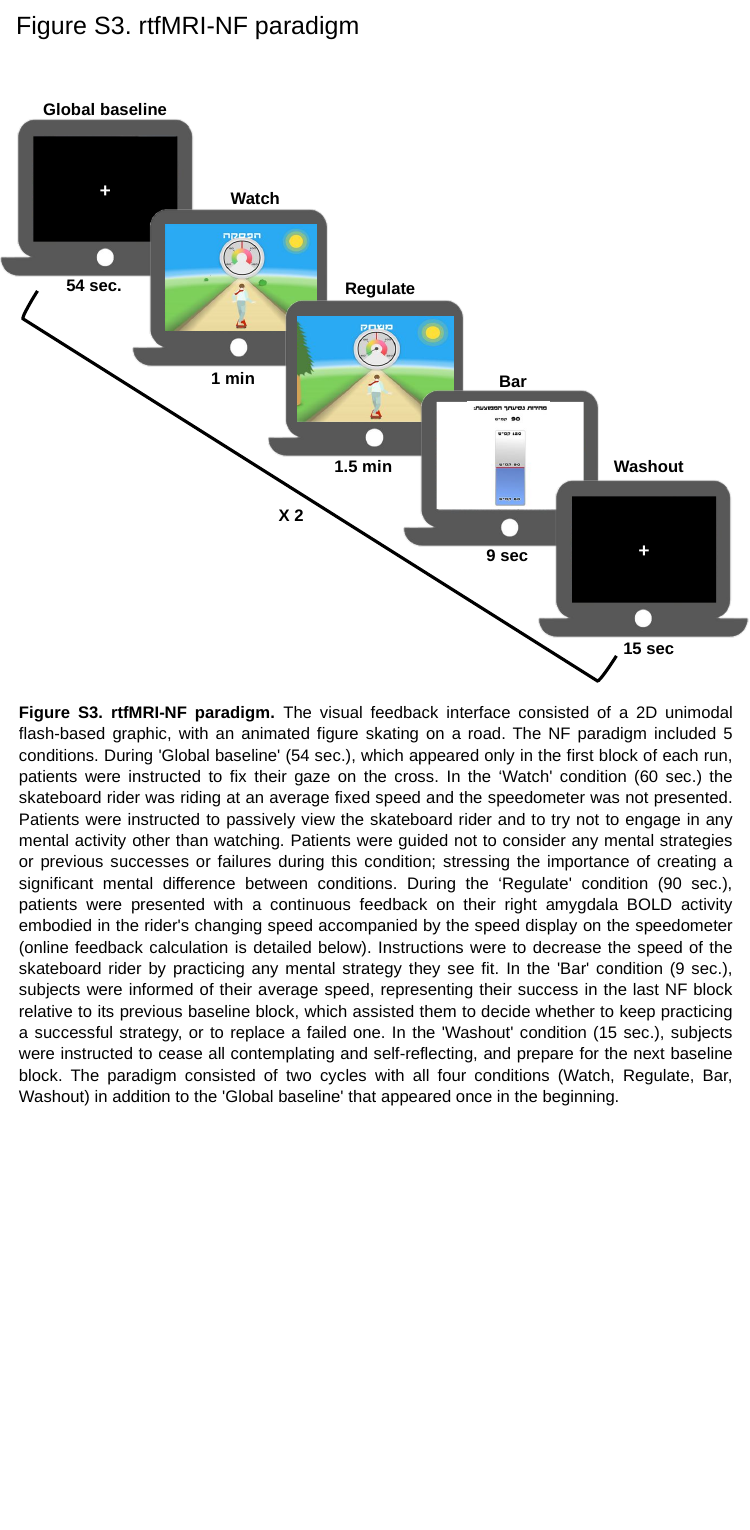

Figure S3. rtfMRI-NF paradigm
Global baseline
+
Watch
54 sec.
Regulate
1 min
Bar
1.5 min
Washout
+
X 2
9 sec
15 sec
Figure S3. rtfMRI-NF paradigm. The visual feedback interface consisted of a 2D unimodal flash-based graphic, with an animated figure skating on a road. The NF paradigm included 5 conditions. During 'Global baseline' (54 sec.), which appeared only in the first block of each run, patients were instructed to fix their gaze on the cross. In the ‘Watch' condition (60 sec.) the skateboard rider was riding at an average fixed speed and the speedometer was not presented. Patients were instructed to passively view the skateboard rider and to try not to engage in any mental activity other than watching. Patients were guided not to consider any mental strategies or previous successes or failures during this condition; stressing the importance of creating a significant mental difference between conditions. During the ‘Regulate' condition (90 sec.), patients were presented with a continuous feedback on their right amygdala BOLD activity embodied in the rider's changing speed accompanied by the speed display on the speedometer (online feedback calculation is detailed below). Instructions were to decrease the speed of the skateboard rider by practicing any mental strategy they see fit. In the 'Bar' condition (9 sec.), subjects were informed of their average speed, representing their success in the last NF block relative to its previous baseline block, which assisted them to decide whether to keep practicing a successful strategy, or to replace a failed one. In the 'Washout' condition (15 sec.), subjects were instructed to cease all contemplating and self-reflecting, and prepare for the next baseline block. The paradigm consisted of two cycles with all four conditions (Watch, Regulate, Bar, Washout) in addition to the 'Global baseline' that appeared once in the beginning.
