# Supplementary material for: Amygdala electrical-finger-print (AmygEFP) NeuroFeedback guided by individually-tailored Trauma script for post-traumatic stress disorder: Proof-of-concept
Source: Neuroimage Clin. 2021 Oct 15;32:102859. doi: 10.1016/j.nicl.2021.102859 (PMC8551212; doi:10.1016/j.nicl.2021.102859)
Supplement: Supplementary data 5 [file mmc5.pptx]

## Slide 1
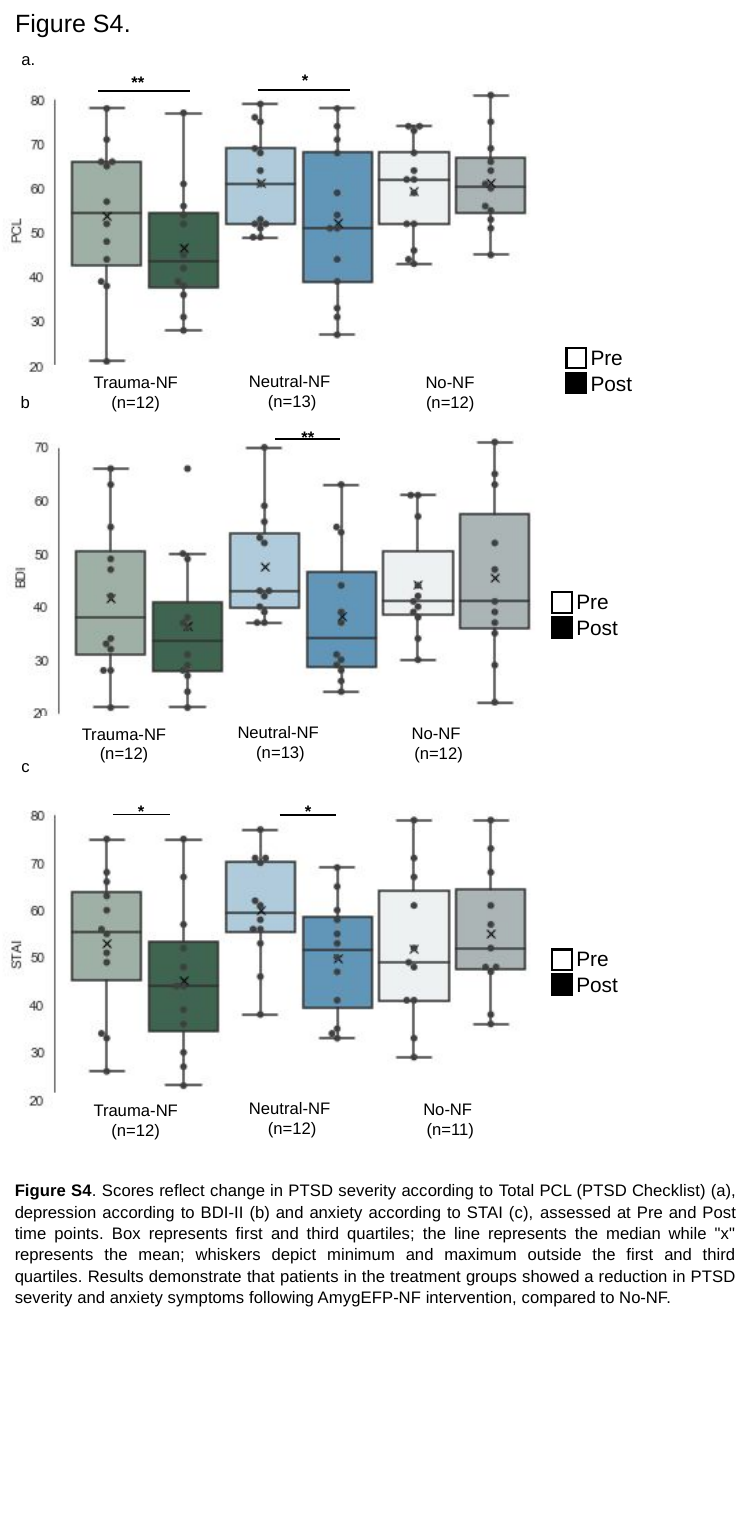

Figure S4.
a.
*
**
Pre
Post
Neutral-NF
(n=13)
No-NF
(n=12)
Trauma-NF (n=12)
b
**
Pre
Post
Neutral-NF
(n=13)
No-NF
(n=12)
Trauma-NF (n=12)
c
*
*
Pre
Post
Neutral-NF
(n=12)
No-NF
(n=11)
Trauma-NF (n=12)
Figure S4. Scores reflect change in PTSD severity according to Total PCL (PTSD Checklist) (a), depression according to BDI-II (b) and anxiety according to STAI (c), assessed at Pre and Post time points. Box represents first and third quartiles; the line represents the median while "x" represents the mean; whiskers depict minimum and maximum outside the first and third quartiles. Results demonstrate that patients in the treatment groups showed a reduction in PTSD severity and anxiety symptoms following AmygEFP-NF intervention, compared to No-NF.
