# Supplementary material for: Amygdala electrical-finger-print (AmygEFP) NeuroFeedback guided by individually-tailored Trauma script for post-traumatic stress disorder: Proof-of-concept
Source: Neuroimage Clin. 2021 Oct 15;32:102859. doi: 10.1016/j.nicl.2021.102859 (PMC8551212; doi:10.1016/j.nicl.2021.102859)
Supplement: Supplementary data 6 [file mmc6.pptx]

## Slide 1
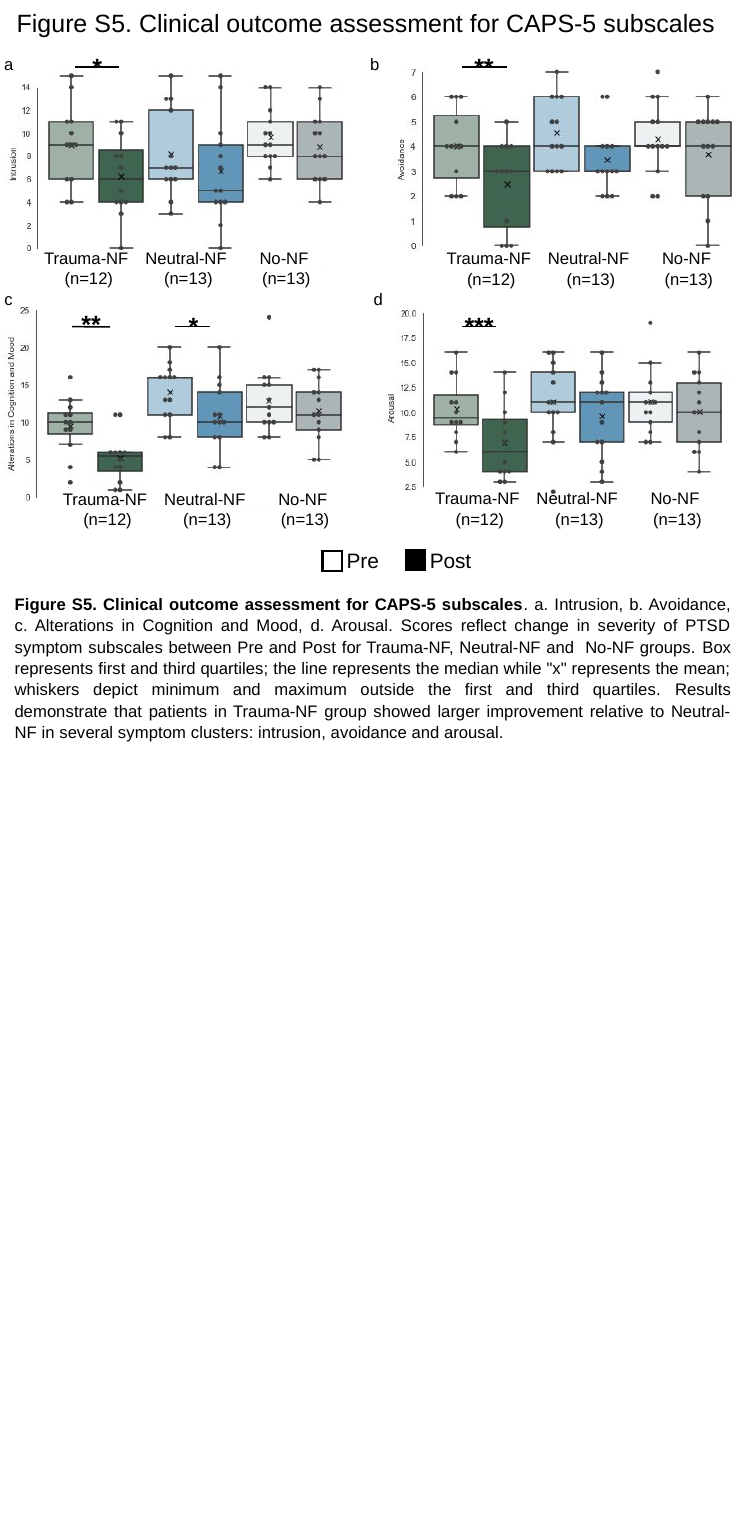

Figure S5. Clinical outcome assessment for CAPS-5 subscales
**
*
a
b
Trauma-NF
(n=12)
Neutral-NF
(n=13)
No-NF
(n=13)
Trauma-NF
(n=12)
Neutral-NF
(n=13)
No-NF
(n=13)
c
d
**
***
*
Trauma-NF
(n=12)
Neutral-NF
(n=13)
No-NF
(n=13)
Trauma-NF
(n=12)
Neutral-NF
(n=13)
No-NF
(n=13)
Pre
Post
Figure S5. Clinical outcome assessment for CAPS-5 subscales. a. Intrusion, b. Avoidance, c. Alterations in Cognition and Mood, d. Arousal. Scores reflect change in severity of PTSD symptom subscales between Pre and Post for Trauma-NF, Neutral-NF and No-NF groups. Box represents first and third quartiles; the line represents the median while "x" represents the mean; whiskers depict minimum and maximum outside the first and third quartiles. Results demonstrate that patients in Trauma-NF group showed larger improvement relative to Neutral-NF in several symptom clusters: intrusion, avoidance and arousal.
