# Supplementary material for: Amygdala electrical-finger-print (AmygEFP) NeuroFeedback guided by individually-tailored Trauma script for post-traumatic stress disorder: Proof-of-concept
Source: Neuroimage Clin. 2021 Oct 15;32:102859. doi: 10.1016/j.nicl.2021.102859 (PMC8551212; doi:10.1016/j.nicl.2021.102859)
Supplement: Supplementary data 7 [file mmc7.pptx]

## Slide 1
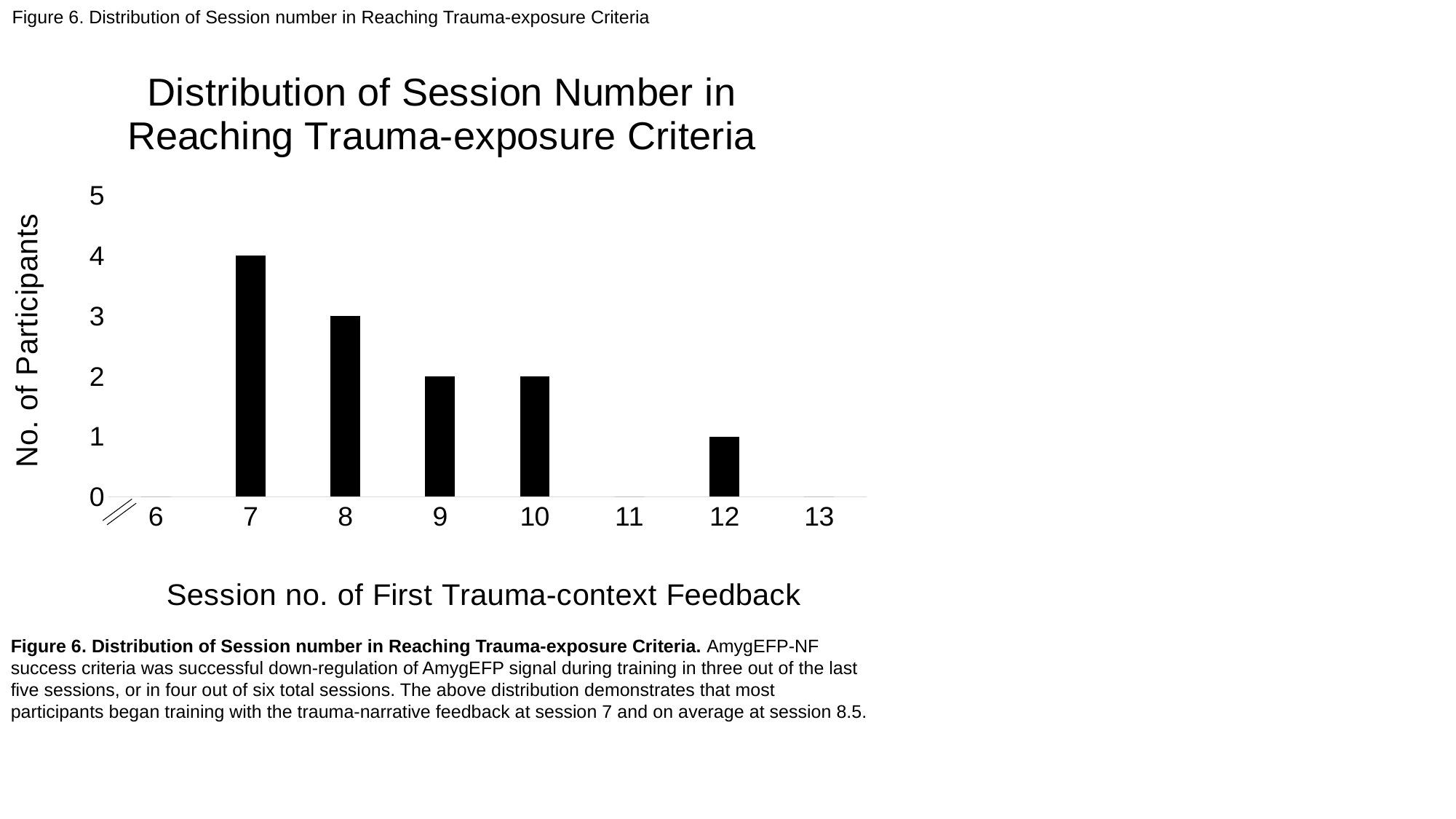

Figure 6. Distribution of Session number in Reaching Trauma-exposure Criteria
### Chart: Distribution of Session Number in Reaching Trauma-exposure Criteria
| Category | No. of Participants |
|---|---|
| 6 | 0.0 |
| 7 | 4.0 |
| 8 | 3.0 |
| 9 | 2.0 |
| 10 | 2.0 |
| 11 | 0.0 |
| 12 | 1.0 |
| 13 | 0.0 |Figure 6. Distribution of Session number in Reaching Trauma-exposure Criteria. AmygEFP-NF success criteria was successful down-regulation of AmygEFP signal during training in three out of the last five sessions, or in four out of six total sessions. The above distribution demonstrates that most participants began training with the trauma-narrative feedback at session 7 and on average at session 8.5.
