# Supplementary material for: Amygdala electrical-finger-print (AmygEFP) NeuroFeedback guided by individually-tailored Trauma script for post-traumatic stress disorder: Proof-of-concept
Source: Neuroimage Clin. 2021 Oct 15;32:102859. doi: 10.1016/j.nicl.2021.102859 (PMC8551212; doi:10.1016/j.nicl.2021.102859)
Supplement: Supplementary data 8 [file mmc8.pptx]

## Slide 1
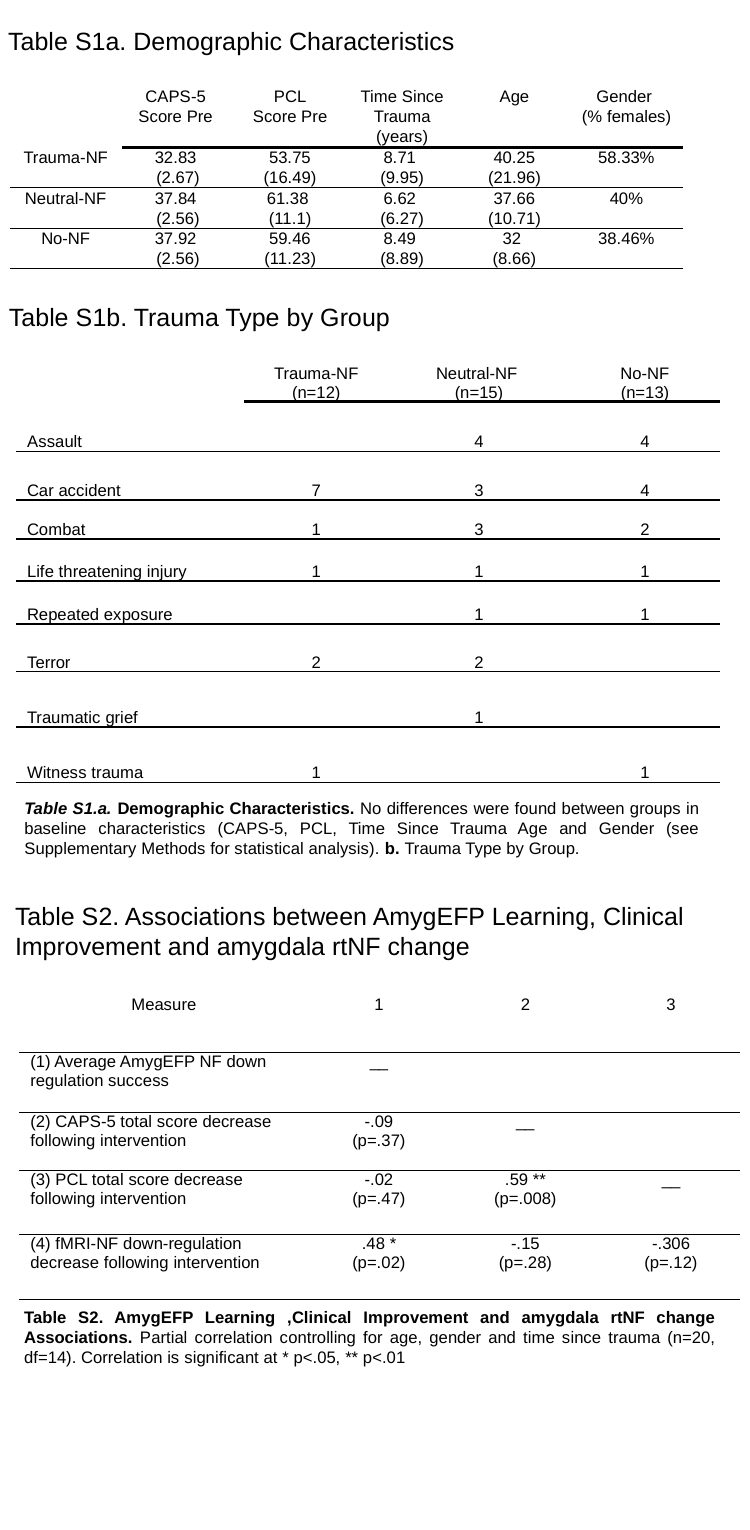

Table S1a. Demographic Characteristics
| | CAPS-5 Score Pre | PCL Score Pre | Time Since Trauma (years) | Age | Gender (% females) |
| --- | --- | --- | --- | --- | --- |
| Trauma-NF | 32.83 (2.67) | 53.75 (16.49) | 8.71 (9.95) | 40.25 (21.96) | 58.33% |
| Neutral-NF | 37.84 (2.56) | 61.38 (11.1) | 6.62 (6.27) | 37.66 (10.71) | 40% |
| No-NF | 37.92 (2.56) | 59.46 (11.23) | 8.49 (8.89) | 32 (8.66) | 38.46% |
Table S1b. Trauma Type by Group
| | Trauma-NF (n=12) | Neutral-NF (n=15) | No-NF (n=13) |
| --- | --- | --- | --- |
| Assault | | 4 | 4 |
| Car accident | 7 | 3 | 4 |
| Combat | 1 | 3 | 2 |
| Life threatening injury | 1 | 1 | 1 |
| Repeated exposure | | 1 | 1 |
| Terror | 2 | 2 | |
| Traumatic grief | | 1 | |
| Witness trauma | 1 | | 1 |
Table S1.a. Demographic Characteristics. No differences were found between groups in baseline characteristics (CAPS-5, PCL, Time Since Trauma Age and Gender (see Supplementary Methods for statistical analysis). b. Trauma Type by Group.
Table S2. Associations between AmygEFP Learning, Clinical Improvement and amygdala rtNF change
| Measure | 1 | 2 | 3 |
| --- | --- | --- | --- |
| (1) Average AmygEFP NF down regulation success | \_\_ | | |
| (2) CAPS-5 total score decrease following intervention | -.09 (p=.37) | \_\_ | |
| (3) PCL total score decrease following intervention | -.02 (p=.47) | .59 \*\* (p=.008) | \_\_ |
| (4) fMRI-NF down-regulation decrease following intervention | .48 \* (p=.02) | -.15 (p=.28) | -.306 (p=.12) |
Table S2. AmygEFP Learning ,Clinical Improvement and amygdala rtNF change Associations. Partial correlation controlling for age, gender and time since trauma (n=20, df=14). Correlation is significant at * p<.05, ** p<.01
