# Supplementary material for: Amygdala electrical-finger-print (AmygEFP) NeuroFeedback guided by individually-tailored Trauma script for post-traumatic stress disorder: Proof-of-concept
Source: Neuroimage Clin. 2021 Oct 15;32:102859. doi: 10.1016/j.nicl.2021.102859 (PMC8551212; doi:10.1016/j.nicl.2021.102859)
Supplement: Supplementary data 9 [file mmc9.pptx]

## Slide 1
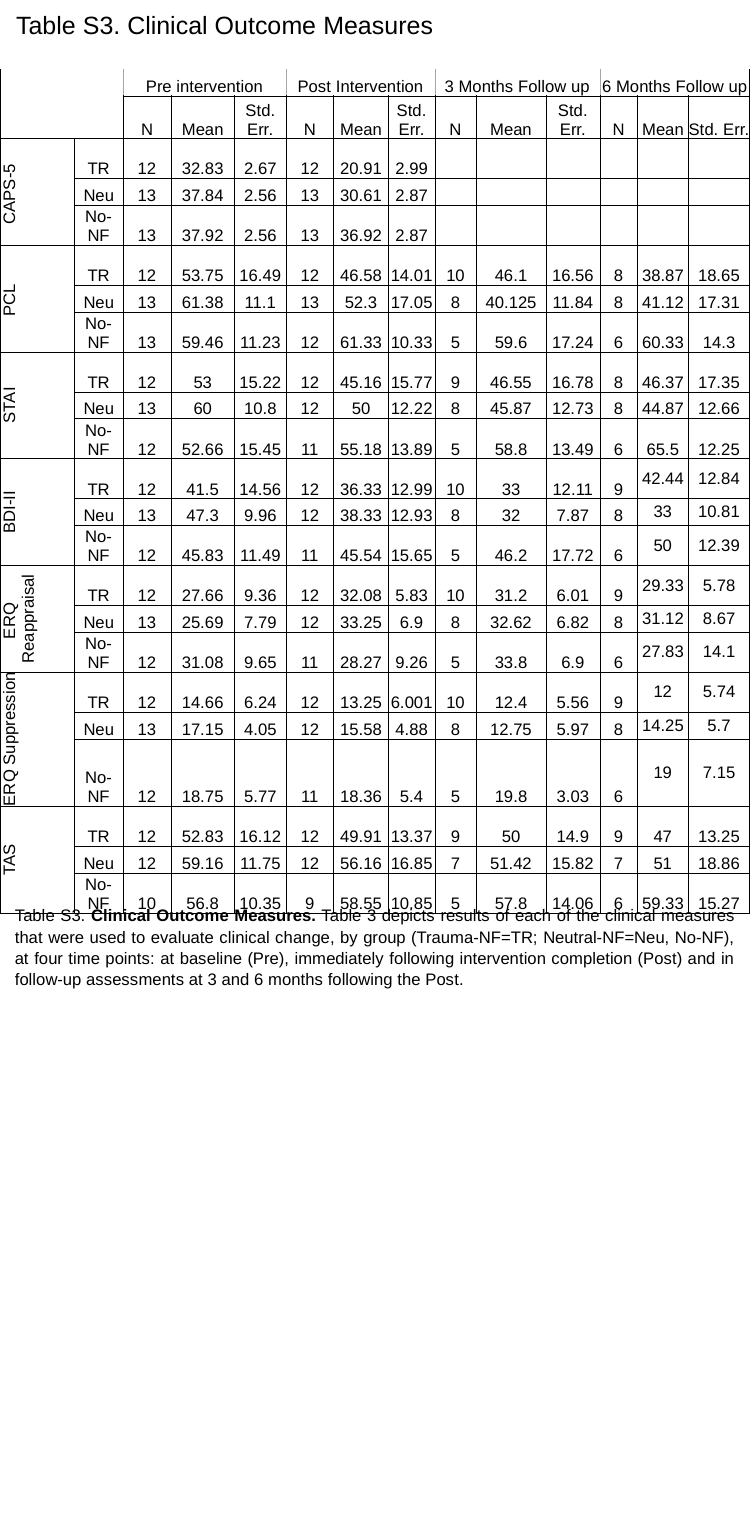

Table S3. Clinical Outcome Measures
| | | Pre intervention | | | Post Intervention | | | 3 Months Follow up | | | 6 Months Follow up | | |
| --- | --- | --- | --- | --- | --- | --- | --- | --- | --- | --- | --- | --- | --- |
| | | N | Mean | Std. Err. | N | Mean | Std. Err. | N | Mean | Std. Err. | N | Mean | Std. Err. |
| CAPS-5 | TR | 12 | 32.83 | 2.67 | 12 | 20.91 | 2.99 | | | | | | |
| | Neu | 13 | 37.84 | 2.56 | 13 | 30.61 | 2.87 | | | | | | |
| | No-NF | 13 | 37.92 | 2.56 | 13 | 36.92 | 2.87 | | | | | | |
| PCL | TR | 12 | 53.75 | 16.49 | 12 | 46.58 | 14.01 | 10 | 46.1 | 16.56 | 8 | 38.87 | 18.65 |
| | Neu | 13 | 61.38 | 11.1 | 13 | 52.3 | 17.05 | 8 | 40.125 | 11.84 | 8 | 41.12 | 17.31 |
| | No-NF | 13 | 59.46 | 11.23 | 12 | 61.33 | 10.33 | 5 | 59.6 | 17.24 | 6 | 60.33 | 14.3 |
| STAI | TR | 12 | 53 | 15.22 | 12 | 45.16 | 15.77 | 9 | 46.55 | 16.78 | 8 | 46.37 | 17.35 |
| | Neu | 13 | 60 | 10.8 | 12 | 50 | 12.22 | 8 | 45.87 | 12.73 | 8 | 44.87 | 12.66 |
| | No-NF | 12 | 52.66 | 15.45 | 11 | 55.18 | 13.89 | 5 | 58.8 | 13.49 | 6 | 65.5 | 12.25 |
| BDI-II | TR | 12 | 41.5 | 14.56 | 12 | 36.33 | 12.99 | 10 | 33 | 12.11 | 9 | 42.44 | 12.84 |
| | Neu | 13 | 47.3 | 9.96 | 12 | 38.33 | 12.93 | 8 | 32 | 7.87 | 8 | 33 | 10.81 |
| | No-NF | 12 | 45.83 | 11.49 | 11 | 45.54 | 15.65 | 5 | 46.2 | 17.72 | 6 | 50 | 12.39 |
| ERQ Reappraisal | TR | 12 | 27.66 | 9.36 | 12 | 32.08 | 5.83 | 10 | 31.2 | 6.01 | 9 | 29.33 | 5.78 |
| | Neu | 13 | 25.69 | 7.79 | 12 | 33.25 | 6.9 | 8 | 32.62 | 6.82 | 8 | 31.12 | 8.67 |
| | No-NF | 12 | 31.08 | 9.65 | 11 | 28.27 | 9.26 | 5 | 33.8 | 6.9 | 6 | 27.83 | 14.1 |
| ERQ Suppression | TR | 12 | 14.66 | 6.24 | 12 | 13.25 | 6.001 | 10 | 12.4 | 5.56 | 9 | 12 | 5.74 |
| | Neu | 13 | 17.15 | 4.05 | 12 | 15.58 | 4.88 | 8 | 12.75 | 5.97 | 8 | 14.25 | 5.7 |
| | No-NF | 12 | 18.75 | 5.77 | 11 | 18.36 | 5.4 | 5 | 19.8 | 3.03 | 6 | 19 | 7.15 |
| TAS | TR | 12 | 52.83 | 16.12 | 12 | 49.91 | 13.37 | 9 | 50 | 14.9 | 9 | 47 | 13.25 |
| | Neu | 12 | 59.16 | 11.75 | 12 | 56.16 | 16.85 | 7 | 51.42 | 15.82 | 7 | 51 | 18.86 |
| | No-NF | 10 | 56.8 | 10.35 | 9 | 58.55 | 10.85 | 5 | 57.8 | 14.06 | 6 | 59.33 | 15.27 |
Table S3. Clinical Outcome Measures. Table 3 depicts results of each of the clinical measures that were used to evaluate clinical change, by group (Trauma-NF=TR; Neutral-NF=Neu, No-NF), at four time points: at baseline (Pre), immediately following intervention completion (Post) and in follow-up assessments at 3 and 6 months following the Post.
