# Supplementary material for: Amygdala electrical-finger-print (AmygEFP) NeuroFeedback guided by individually-tailored Trauma script for post-traumatic stress disorder: Proof-of-concept
Source: Neuroimage Clin. 2021 Oct 15;32:102859. doi: 10.1016/j.nicl.2021.102859 (PMC8551212; doi:10.1016/j.nicl.2021.102859)
Supplement: Supplementary data 10 [file mmc10.pptx]

## Slide 1
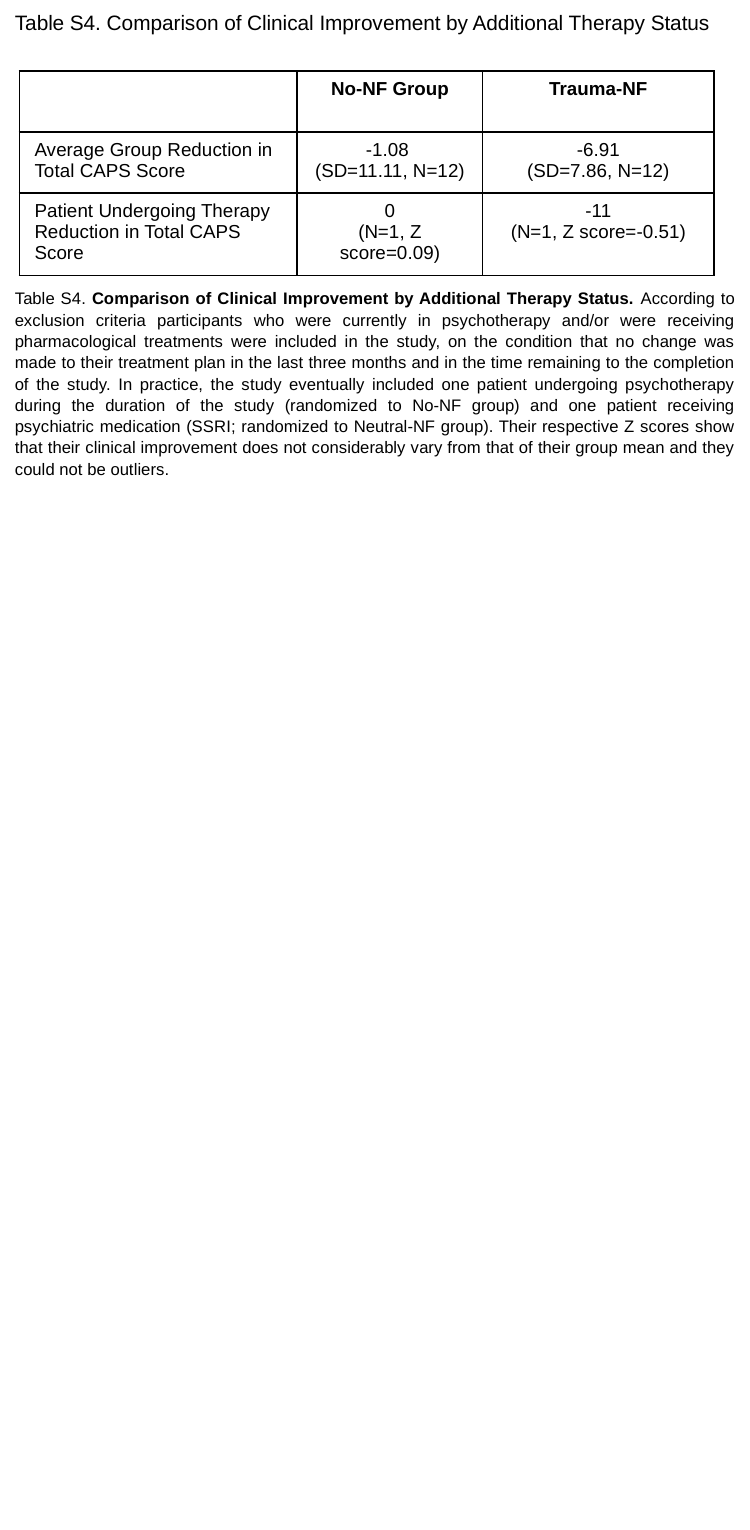

Table S4. Comparison of Clinical Improvement by Additional Therapy Status
| | No-NF Group | Trauma-NF |
| --- | --- | --- |
| Average Group Reduction in Total CAPS Score | -1.08 (SD=11.11, N=12) | -6.91 (SD=7.86, N=12) |
| Patient Undergoing Therapy Reduction in Total CAPS Score | 0 (N=1, Z score=0.09) | -11 (N=1, Z score=-0.51) |
Table S4. Comparison of Clinical Improvement by Additional Therapy Status. According to exclusion criteria participants who were currently in psychotherapy and/or were receiving pharmacological treatments were included in the study, on the condition that no change was made to their treatment plan in the last three months and in the time remaining to the completion of the study. In practice, the study eventually included one patient undergoing psychotherapy during the duration of the study (randomized to No-NF group) and one patient receiving psychiatric medication (SSRI; randomized to Neutral-NF group). Their respective Z scores show that their clinical improvement does not considerably vary from that of their group mean and they could not be outliers.
